# 40 Years of diffusion MRI in the brain: From history to emerging frontiers

**DOI:** 10.1162/IMAG.a.1365

**Published:** 2026-09-15

**Authors:** Denis Le Bihan

**Affiliations:** NeuroSpin, CEA, CEA-Saclay Center, Paris-Saclay University, Gif-sur-Yvette, France

**Keywords:** diffusion MRI, brain microstructure, acute stroke, DTI, tractography, IVIM, DfMRI, MRE, connectome, brain biomechanics

## Abstract

Diffusion MRI transformed brain imaging by making MRI sensitive not only to anatomy and spin-relaxation contrast, but also to micrometer-scale water displacements over diffusion times of a few tens of milliseconds. The resulting signal is exquisitely sensitive to tissue organization, because cell membranes, myelin, axonal packing, orientation dispersion, exchange, as well as perfusion-related incoherent fluid motion all modulate the displacement distribution sampled by diffusing water molecules. In the brain, this sensitivity proved historically decisive in three domains. First, diffusion-weighted MRI enabled the early detection of acute ischemia by revealing tissue injury before it became apparent on conventional structural imaging, thereby supporting timely treatment and improving outcomes for millions of patients worldwide. Second, the strong orientational order of white matter produces diffusion anisotropy, which can be explored through a diffusion tensor imaging (DTI) framework, ultimately leading to the reconstruction of large-scale structural pathways and connectomes in vivo, relevant to psychiatric disorders. Third, advances in gradient hardware, multi-shell acquisition, and biophysical modeling extended the field beyond the original apparent diffusion coefficient (ADC) concept, revealing tissue microstructure through time-dependent and multi-compartment diffusion behavior, with applications to brain development and myelination disorders, neurodegeneration, and neurosurgery. This article reviews the brain-centered evolution of diffusion MRI from its physical foundations to its forward-looking frontiers. Throughout, the central argument is that diffusion MRI derives its power from sensitivity to microstructure, whereas its interpretation depends critically on model assumptions and acquisition design.

## 1 Introduction: Why the Brain Became the Natural Organ for Diffusion MRI

MRI entered clinical medicine as a fundamentally macroscopic modality. Its native contrasts, proton density, T1, T2, and later T2*, were immensely successful for depicting soft-tissue anatomy, edema, hemorrhage, tumor, and inflammation, but they remained indirect contrasts of tissue state rather than explicit measurements of tissue geometry. Diffusion MRI altered this situation by encoding a physical process that exists independently of the scanner: the thermally driven displacement of water molecules. Because the root-mean-square displacement during typical diffusion times is measured in micrometers rather than millimeters, diffusion weighting couples an image voxel to sub-voxel tissue organization and thereby opens MRI to microstructural inference (Le Bihan, 1995).

The brain became the natural proving ground for this strategy for technical and clinical reasons. Diffusion MRI was initially tested in a small number of patients with liver tumors, but substantial motion artifacts compromised results in the abdomen. Because the head is comparatively less affected by motion than the abdomen, diffusion MRI was then tested in patients with brain tumors, with striking results. Acute stroke, however, gave diffusion MRI its first compelling clinical indication: it enabled objective diagnosis at the earliest stage by detecting reduced water mobility in hypoxic tissue associated with cytotoxic edema, thereby opening the way to clinical trials for emergency thrombolytic therapies (Chien et al., 1992; Moseley, Cohen, Mintorovitch, et al., 1990; Warach et al., 1992).

From a signal-theory perspective, diffusion MRI is not a single biomarker but an acquisition-and-model framework. The measured attenuation depends on the MRI gradient waveform, resulting diffusion sensitivity, diffusion time, and the extent to which the chosen biophysical model matches tissue reality. The same reduction in apparent diffusivity may arise from cytotoxic edema, increased cellular packing, altered membrane permeability, orientation dispersion, partial volume with CSF, or vascular effects. Brain diffusion MRI must, therefore, be interpreted simultaneously through physics, biology, image contrast, and inverse problem theory, making it a rich and powerful imaging modality (Jelescu & Budde, 2017; Le Bihan, 1995; Novikov et al., 2019).

The goal of this article is, therefore, not simply to provide a detailed technical account or an exhaustive review of applications, which are available elsewhere, including other articles of this special issue. Rather, it is to articulate how the technical logic linking diffusion MRI signal formation to biological and clinical interpretation emerged and evolved over the past 40 years, from its historical foundations to forward looking concepts that are only beginning to take shape.

## 2 Historical Foundations and the Birth of Brain Diffusion MRI

The conceptual roots of diffusion MRI reach back to classical physics and the first decades of NMR. Einstein’s treatment of Brownian motion established that random molecular displacements give rise to diffusion and linked the mean-squared displacement to the diffusion coefficient, transforming an apparently disordered microscopic phenomenon into a quantitative physical law (Einstein, 1905). In the NMR era, diffusion was regarded as a source of nuisance, causing signal attenuation and spectral line broadening in the early NMR systems affected by substantial magnetic field inhomogeneities. Hahn’s spin echo showed how dephasing and rephasing could be manipulated experimentally, while Carr and Purcell, and later Torrey, formalized how diffusion in the presence of gradients modifies signal evolution (Carr & Purcell, 1954; Hahn, 1950; Torrey, 1956). The decisive experimental step came from Stejskal and Tanner, who showed that paired diffusion-sensitizing gradient pulses could encode translational motion in a controlled way through diffusion-driven signal attenuation caused by phase-shift dispersion from molecular displacements within the inhomogeneous magnetic field generated by the gradient pulses (Stejskal & Tanner, 1965).

For one-dimensional free diffusion, Einstein showed that the mean-squared displacement grows linearly with diffusion time, T_d_, according to:

$$\langle {\text{X}}^{2}\rangle =2D\;{\text{T}}_{\text{d}},$$ (1)

where 〈X²〉 is the mean-squared diffusion distance along one spatial direction. For water at body temperature, D is on the order of 3 × 10⁻³ mm² s⁻¹ in free solution, so during T_d_ ≈ 50 ms, the characteristic displacement is on the order of 10–20 µm, that is, comparable with cellular dimensions in brain tissue. This simple scaling argument is the conceptual bridge that makes diffusion MRI a sub-voxel probe of microstructure (Le Bihan, 1995).

With Gaussian (free) diffusion, the normalized signal attenuation, S(b)/S(0), obeys in the ideal narrow-pulse Stejskal–Tanner limit using a pair of gradients of amplitude g, duration δ, and separation Δ, with δ << Δ is:

$$\text{S}\left(\text{b}\right)/\text{S}\left(0\right)=\text{exp}\left[-\gamma^{2}{\text{ g}}^{2}\delta^{2}\left(\Delta -\delta /3\right)\text{D}\right].$$ (2)

In imaging practice, however, the ideal narrow pulse Stejskal–Tanner equation is insufficient because imaging gradient pulses, crusher gradient pulses, and their cross-terms with the diffusion-encoding gradient pulses can contribute substantially to the diffusion signal attenuation (Cohen et al., 2015; Le Bihan & Breton, 1985; Rashid et al., 2025). These terms must be included in the calculation, which led to the definition of the so-called *b*-*value* (Le Bihan & Breton, 1985) that integrates all relevant gradient pulse terms and quantifies the degree of sensitization of any MRI sequence to diffusion:

S(b)/S(0)=exp(− b D). (3)

**Fig. 1.**
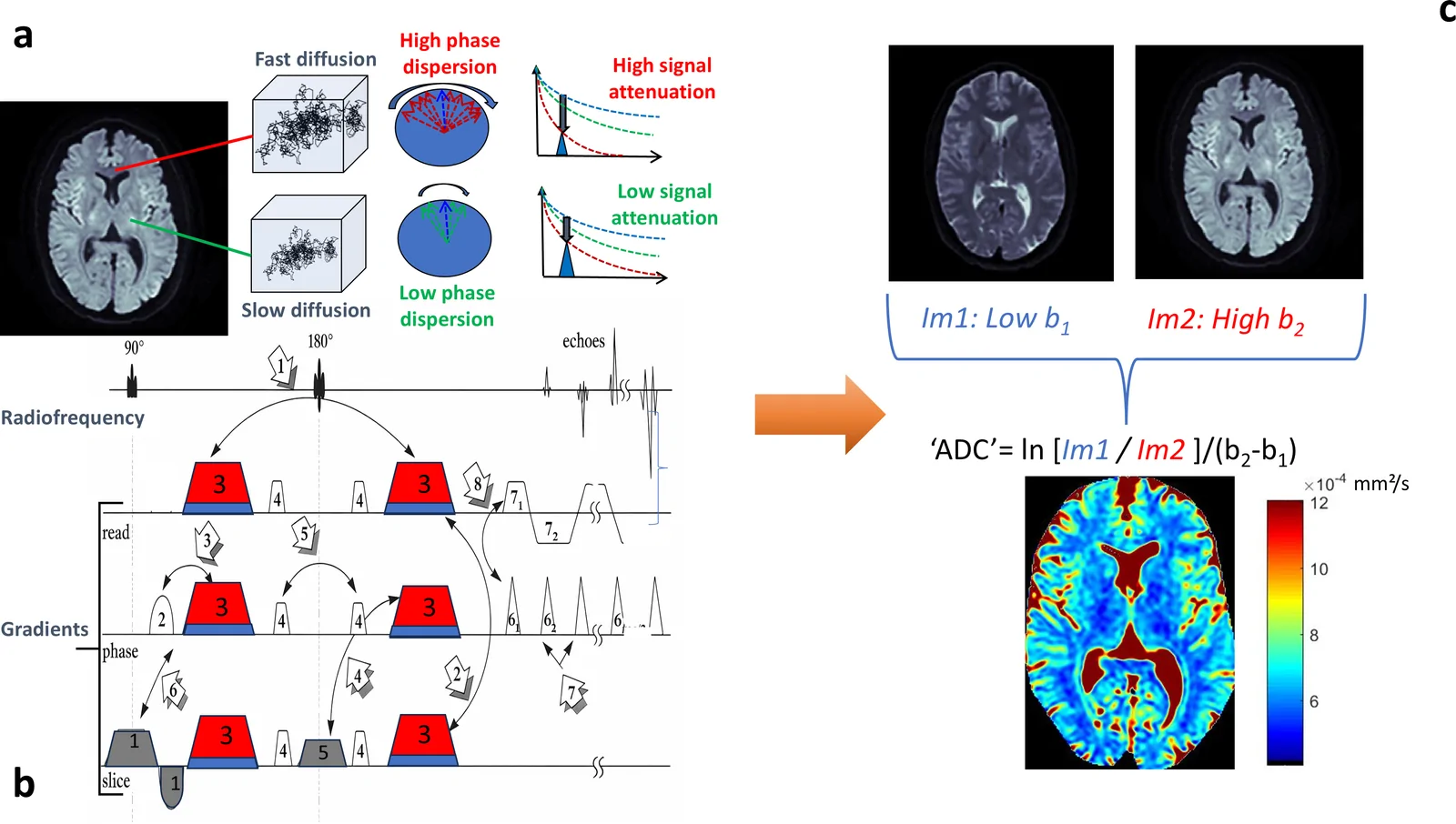
Principle of diffusion MRI. (a) During the presence of magnetic field gradients, the diffusing movement of water molecules results in phase shifts of their magnetization according to their displacement history. Given the extremely large number of molecules present in a voxel and the randomness of such movements, the phase shifts are distributed within each voxel, resulting in a signal loss, more pronounced with fast diffusion than slow diffusion. (b) Typical diffusion-EPI sequence with diffusion-encoding gradient pulses (3; blue, low amplitude; red, high amplitude). The diffusion gradients can be set on either axis or multiple axes to get oblique diffusion-encoding directions, and with different amplitudes according to the desired degree of diffusion sensitivity, reflected in the b-value, which also includes the contribution of all other imaging gradient pulses (1: slice selection and refocusing; 2: pre-dephasing; 4: crushers; 5: slice selection; 6: phase-encoding; 7: readout), independently and through cross-terms (blue boxes with numbers). This also means that the diffusion time and the diffusion-encoding direction depend on the choice of imaging parameters. (c) While the images obtained with this sequence are “diffusion-weighted” as well as T1 and T2 weighted, “pure” diffusion images (ADC maps) are obtained by combining images acquired with two different b-values from Eq. (4).

**Fig. 2.**
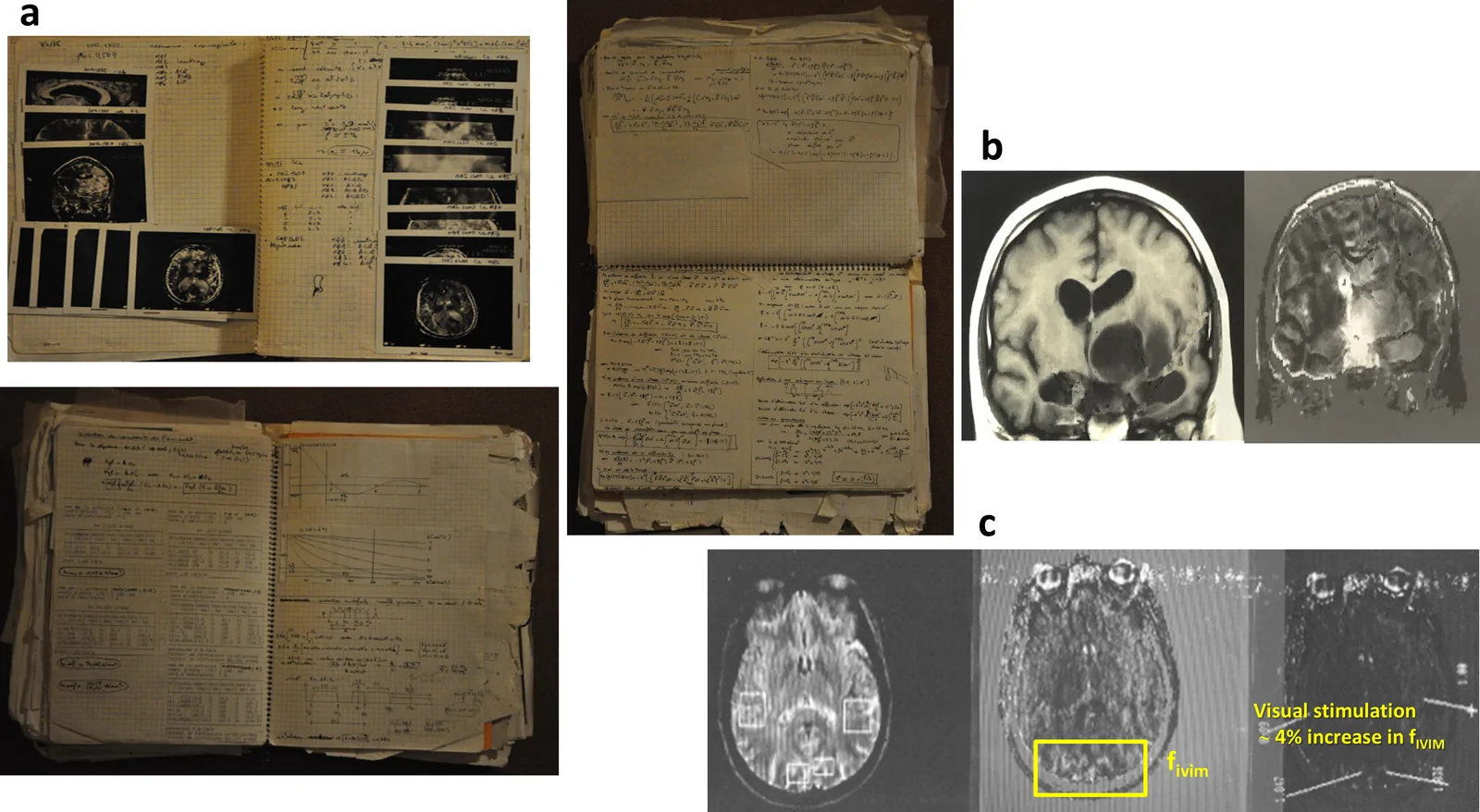
Historical implementation. (a) Copies of the author’s notebook (1986) showing the very first diffusion MR images obtained in patients with brain lesions with calculations providing estimation of perfusion-IVIM and diffusion parameters (including cell size), as well as simulations of IVIM effects and calculations of relevant b-values, which led to the implementation of the IVIM/diffusion MRI sequence. (b) Examples of early diffusion images obtained in a young patient with a cystic tumor. IVIM effects resulting in ventricles from CSF flow patterns as striking. Adapted, in part, with permission of the Radiological Society of North America (RSNA), from Le Bihan et al. (1986). (c) Early attempt of IVIM fMRI (visual stimulation) showing a 4% increase of fIVIM in the visual cortex (copy of the author’s notebook, 1986).

MRI supplied the final ingredient missing from earlier diffusion NMR studies: spatial encoding. Once diffusion weighting could be combined with imaging, diffusion coefficients could be mapped across a tissue volume rather than measured only as a bulk volume average in spectroscopy. The earliest clinical implementations were technically fragile: gradient performance was limited (maximal value of 10 mT/m, restricting b-values to 200 s/mm² or less, magnetic field strength was low (0.35 and 0.5 T), acquisitions were slow (around 8–10 minutes for each b-value), and motion sensitivity was severe (Figs. 1 and 2). In that context, the earliest in vivo demonstrations of diffusion imaging were both conceptual and practical breakthroughs (Le Bihan & Breton, 1985; Le Bihan et al., 1986, 1988).

It is important to notice that the b-value has been a pillar element of diffusion MRI, because it enabled an accurate quantification of diffusion weighting in complete imaging sequences, rather than only in idealized pulse sequences as assumed with Eq. (2). Earlier approaches were important historical steps but had substantial limitations. Wesbey et al. (1984) proposed a semiquantitative method requiring an external reference, such as acetone, and modulation of slice-selection gradients, making it unsuitable for routine clinical use. Taylor and Bushell (1985) used quasi-continuous gradients to estimate diffusion in hen eggs using Hahn’s equations without accounting for imaging-gradient contributions. Merboldt et al. (1985) acquired diffusion images of phantoms with a stimulated-echo (STEAM) sequence but likewise did not incorporate imaging-gradient terms; moreover, STEAM entails an intrinsic 50% signal loss.

Using Eq. (2) in diffusion MRI can substantially underestimate the true b-value and hence overestimate diffusion, because the measured signal is more attenuated than predicted by the idealized expression. In a standard T2-weighted spin-echo sequence, imaging gradients contribute a nonzero baseline b-value (which, hence, cannot be 0, as often reported)., typically around 10–50 s/mm². When diffusion-encoding gradients of, for example, 30 mT/m are added to reach a b-value near 1,000 s/mm², imaging gradients and cross-terms may account for approximately 15% of the total. With diffusion-encoding gradients of about 50 mT/m, the nominal b-value may reach 3,000 s/mm², with imaging-gradient contributions still around 10%. The issue becomes particularly important in multi-shell acquisitions because these contributions vary across shells, causing erroneous diffusion weighting if they are not properly accounted for.

Around the same time, the intravoxel incoherent motion (IVIM) framework generalized diffusion MRI by recognizing that intravoxel microscopic motion includes both molecular diffusion and microvascular flow components (Le Bihan et al., 1986, 1988). These early developments are particularly important for a brain-focused history because they framed diffusion MRI from the outset as a method capable of probing more than static anatomy. In the brain, beyond characterizing tissue microstructure, diffusion MRI more broadly appeared to offer a route to perfusion information and, potentially, brain function through neurovascular coupling (see below; Fig. 2) (Roy & Sherrington, 1890).

A practical turning point was rapid imaging. Diffusion weighting magnifies the impact of bulk motion, and the early slow acquisitions made many organs nearly impossible to image robustly. An early approach used a Steady-State-Free-Precession (SSFP) sequence (Le Bihan, 1988), which allowed a higher signal-to-noise ratio and shorter diffusion times. Quantification remained difficult, however, because diffusion weighting accumulates across complex echo pathways and depends on T1 and T2. Besides, sensitivity to motion persisted. Echo-planar imaging (EPI) reduced vulnerability to patient motion and physiological pulsatility by collapsing acquisition into very short readouts. With Robert Turner and colleagues at General Electric Medical Sytems (GEMS), we showed that EPI effectively combined with IVIM and diffusion methods, setting the stage for clinical neuroimaging applications (Turner et al., 1990). In retrospect, this technical step was as important as the underlying physics. Faster acquisitions, together with stronger gradients supporting EPI, made it possible to acquire multiple diffusion-weighted images up to b = 1,000 s/mm² in approximately 1 minute with substantially reduced motion sensitivity. Without fast acquisition, the brain may still have been a promising target, but the hyperacute stroke applications that defined the field would not have become clinically practical when they did. Furthermore, the gradient hardware made to support EPI also allowed to reach much higher b-values: Suddenly, with a head gradient coil capable of 40 mT/m, it became possible to acquire a set of diffusion-weighted images 16 b-values up to 1,000 s/mm² in just 1 minute and without motion artifacts.

The birth of brain diffusion MRI was, therefore, not a single event but a convergence of physical theory, NMR pulse-sequence design, MRI hardware, and neurological need. By the end of the 1980s, the field had the core ingredients that would dominate the following decades: diffusion-weighted contrast, IVIM as a generalized motion framework, echo-planar acquisition, and the intuition that tissue microstructure could be inferred from measurements made at the much coarser image-voxel scale. The subsequent history of brain diffusion MRI can be read as a sequence of increasingly ambitious claims about what those measurements mean (Le Bihan, 2003, 2024a).

## 3 The Apparent Diffusion Coefficient (ADC) and IVIM Concepts

### 3.1 Apparent diffusion coefficient

The most compact starting point remains Einstein’s relation for one-dimensional free diffusion, where the mean-squared displacement grows linearly with diffusion time according to Eq. (1). In pure water at body temperature, this relation implies micrometer-scale displacements over the tens of milliseconds relevant to MRI. That scale is precisely why diffusion MRI became biologically meaningful. Although voxels are millimeters across, the encoded molecular trajectories sample spaces comparable with cellular and subcellular dimensions. The method is, therefore, sensitive to tissue organization that remains well below nominal image resolution.

It is worth emphasizing, however, that diffusion MRI does not measure a geometrical structure directly; it samples displacement statistics in the inhomogeneous magnetic field generated by the gradient pulses over the diffusion time. The “diffusion coefficient”, D, visible in Eq. (3) depends on barriers, pores, cells, axons, exchange, and orientation distributions, while the “b-value” depends on gradient strength, timing, and waveform. This is why it was decided early on (Le Bihan et al., 1986) to rewrite Eq. (3) using an apparent diffusion coefficient (ADC) (Fig. 1):

S(b)/S(0)=exp(− b ADC). (4)

**Fig. 3.**
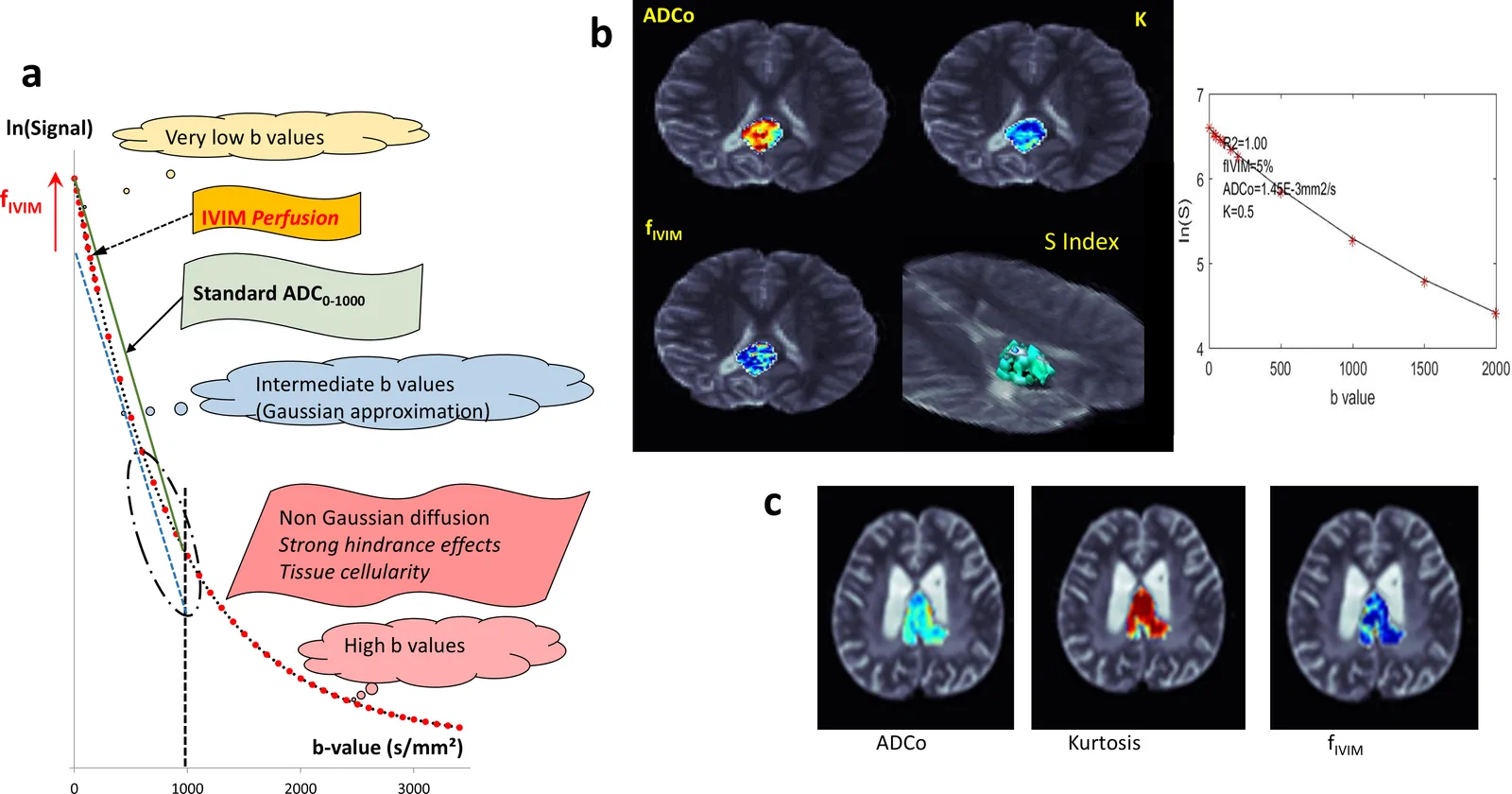
The diffusion-weighted signal behavior. (a) The diffusion-weighted signal attenuation plotted as a function of the b-value reflects a complex, multi-domain behavior. As pseudo-diffusion (D*) is about 10 times higher than diffusion (D), the resulting perfusion-driven IVIM effect is seen as an increase in curvature of the signal decay at low b values and becomes negligible for high b-values (typically above b = 200 s/mm²). The intercept at b = 0 is the flowing blood volume, f_IVIM_. At intermediate b-values, the attenuation is close to a straight line, reflecting quasi-Gaussian diffusion. At high b-value, the attenuation is markedly curved due to non-Gaussian diffusion effects (diffusion hindrance). Because of this curvature, the ADC value highly depends on the choice of b-value, decreasing when b-value increases. It results that the ADC calculated from signals at b = 0 and 1,000 s/mm² (green line) includes both perfusion-driven IVIM effects and some non-Gaussian diffusion effects, deviating from ADCo (blue dotted line), which reflects genuine tissue diffusion when b gets close to 0. This decrease of the ADC with the b-value reflects the increasing contribution of the moderately and highly hindered diffusion sub-compartments, the later dominating the diffusion-weighted image contrast at very high b-values. (b) Example of a young patient with a glioblastoma of the splenium after radio- and chemotherapy. The curved signal decay can be empirically modeled through the IVIM/Kurtosis framework (Eq. (5)), giving estimates of f_IVIM_ (perfusion), ADCo (diffusion at low b-values), and Kurtosis (deviation from a Gaussian behavior due to hindrance effects results linked to cell density). The S-index provides a direct overall capture (no model) of the signal profile compared with a library of signals from reference tissues. In this example, the high ADCo, low f_IVIM_, low K, and low S-index values all point out to the efficacy of the treatment in this part of the lesion (collaboration Mail-Lan Ho, Department of radiology, Nationwide Children Hospital, Columbus, Ohio, USA). (c) Example in a case of radiation necrosis (low ADCo, high K, high f_IVIM_).

Importantly, the ADC, most often estimated from diffusion MRI signals acquired at two b-values (e.g., b as close as possible to 0, however, see above, and b = 1,000 s/mm²) and inverting Eq. (4), is not an intrinsic or effective diffusion coefficient, but an acquisition-dependent parameter summarizing diffusion-related signal attenuation *under specific experimental* conditions. The term *apparent* is essential because diffusion in tissues is not free, but multi-compartmental, hindered, restricted, and heterogeneous. Thus, the ADC reflects an aggregate of coexisting microstructural features rather than a single physical constant, and may also include contributions from other effects such as microvascular pseudo-diffusion (see below). But *apparent* also means that the ADC depends on the measurement conditions, especially the b-value, which must always be reported, but also the diffusion time, a separate parameter that is most often less visible to users (see below). At low-to-intermediate b-values ranges, the signal is dominated by relatively unrestricted or fast-moving water, whereas at higher b-values, these components are attenuated and the signal becomes more influenced by hindered or slowly diffusing water, producing curvature in signal decay. Because ADC varies with b-value, Eq. (4) is misleading: it does not describe true mono-exponential decay, even if a mono-exponential fit might work numerically within at medium range of b-values (e.g., 200–600 s/mm²). In other words, a mono-exponential fit may be used operationally to estimate ADC over a specified b-value range, but the resulting parameter should be interpreted as an acquisition-dependent effective summary rather than evidence for truly Gaussian diffusion, the ADC is not a “Gaussian” model, as sometimes reported. Recognizing this is essential for correct diffusion MRI interpretation (Fig. 3).

The notion of an *ensemble average* at the voxel level further complicates interpretation. A voxel contains a distribution of microscopic environments, and the MRI signal sums over all spins, weighted by their T1 and T2 properties and, therefore, by the sequence TR and TE, in addition to diffusion-driven attenuation. No routine clinical sequence separates these contributions explicitly. Diffusion MRI is powerful precisely because it compresses complex tissue behavior into measurable signal changes, but this compression also entails limited specificity. Two very different tissues can produce similar ADC values if their underlying compartment mixtures differ in one way and converge in another. The interpretive challenge is to design experiments and models that make this many-to-one mapping less ambiguous (Jelescu & Budde, 2017; Jelescu et al., 2016; Le Bihan, 1995).

Another fundamental point is time dependence. The ADC measured at one diffusion time may not match the value measured at another, even when the same b-values are used. As diffusion time increases, water molecules probe larger portions of the tissue environment and encounter more barriers or opportunities for exchange (Le Bihan, 1995; Novikov et al., 2019). Early diffusion MRI used weak gradients and, therefore, required long diffusion times (>70 ms) for sufficient encoding. This limitation provided strong tissue contrast and helped establish diffusion MRI clinically; at much shorter diffusion times, contrast would have been weaker, reflecting mainly viscosity, and diffusion MRI might have not developed. Furthermore, in standard clinical sequences, the effective diffusion time is only approximate because imaging and diffusion-encoding gradients are mixed. Today, stronger gradients and dedicated diffusion-MRI sequences, such as oscillating-gradient spin echo (OGSE), permit shorter diffusion times and reveal additional tissue-specific information through diffusion-time-dependent water behavior (Does et al., 2003; Zhu et al., 2025). Time dependence is, therefore, not a nuisance but a resource: it contains information about spatial scales and compartment sizes.

### 3.2 Perfusion and IVIM

Another contributor to ADC is blood microcirculation, particularly at very low b-values. Under appropriate conditions, blood flow through quasi-randomly oriented capillary segments can attenuate the diffusion-weighted MRI signal within the IVIM framework. This insight complicated interpretation, but it also opened a path toward a broader physiology of microscopic motion in brain tissue (Le Bihan, 2019; Le Bihan & Turner, 1992; Le Bihan et al., 1988). Although it operates on a very different physical scale, blood water moving through capillary networks arranged in a quasi-random spatial pattern can be described as a random walk, pseudo-diffusion process, as blood changes direction repeatedly from one capillary segment to the next, making Einstein’s diffusion model conceptually applicable. The main difference lies in scale: At the molecular scale, individual water-motion steps are nanometer scale, whereas net molecular displacements over MRI diffusion times are micrometer scale; capillary pseudo-diffusion involves segment lengths of tens of micrometers. Because diffusion coefficients reflect both displacement distance and velocity, rapid molecular motion over short distances and slower blood flow over longer distances can yield coefficients differing by only about one order of magnitude in brain tissue. The tissue diffusion coefficient D is typically around 1 × 10⁻³ mm²/s, whereas the pseudo-diffusion coefficient D* associated with capillary flow is often around 10 × 10⁻³ mm²/s

This proximity means that both diffusion and pseudo-diffusion contribute to the ADC, while also allowing their separation in principle. With the diffusion-perfusion IVIM model, the signal attenuation is written as

$$\begin{array}{l} \text{S}\left(\text{b}\right)/\text{S}\left(0\right)={\text{f}}_{\text{IVIM}}\text{ exp}\left[-\text{b}\left({\text{D}}^{*}+\text{D}\_\text{blood}\right)\right]\\ \;\;\;\;\;\;\;\;\;\;\;\;\;\;\;\;\;\;\;\;\;+\left(1-{\text{ f}}_{\text{IVIM}}\right)\text{ exp}\left(-\text{bD}\right), \end{array}$$ (5)

where f_IVIM_ is the flowing-blood fraction, D the tissue diffusion coefficient, D_blood the diffusion coefficient of water in blood, and D* a pseudo-diffusion coefficient reflecting incoherent capillary flow. In the brain, D* is typically much larger than D, so the IVIM contribution is concentrated at very low b-values, but remains difficult to separate robustly from diffusion because f_IVIM_ is small (around a few percent) (Fig. 3) (Iima & Le Bihan, 2016; Le Bihan, 2019; Le Bihan & Turner, 1992; Le Bihan et al., 1988). Especially, accurate b-value calculation is critical, including imaging gradients and cross-terms with diffusion-encoding gradients, as these effects are far from negligible at low b-values (Cohen et al., 2015; Rashid et al., 2025).

The conceptual importance of IVIM for brain neuroimaging has been substantial, broadening diffusion MRI from a method of tissue microstructure toward a more general method capable of quantitative imaging of perfusion parameters (e.g., blood volume and blood flow through f_IVIM_ and D*) without the need for exogenous tracers or contrast agents (Iima & Le Bihan, 2016; Le Bihan, 2019). This broader perspective also contributed to attempts to use IVIM MRI for brain functional imaging (Fig. 2) and to study CSF-related motion (see below).

## 4 Acute Ischemia and Stroke: The First Transformative Brain Application

The first application that irreversibly changed the status of diffusion MRI in medical imaging was acute cerebral ischemia. In hyperacute stroke, the biochemical cascade begins within minutes of arterial occlusion. Energy failure disrupts ion gradients, Na+/K+-ATPase activity collapses, and water redistributes between extracellular and intracellular compartments. Conventional structural MRI and CT do not immediately register these microscopic events with adequate sensitivity, but diffusion-weighted MRI does. In animal studies, Moseley and colleagues showed that diffusion changes appear extraordinarily early after ischemia, preceding many conventional imaging abnormalities and closely tracking the evolving lesion (Moseley, Cohen, Mintorovitch, et al., 1990; Moseley, Kucharczyk, Mintorovitch, et al., 1990).

Translation to human patients depended on fast imaging, and EPI methods were decisive in allowing lesions to be detected within a clinically relevant time frame (Warach et al., 1992). This observation was revolutionary for two reasons, altering neuroradiology and stroke management alike. First, it established that diffusion MRI was not merely a subtle tissue-characterization method; it could detect a neurological emergency at a stage when treatment decisions were most urgent, helping to enable clinical trials of thrombolytic therapies that could spare patients from devastating outcomes. Second, it provided strong empirical evidence that diffusion MRI was sensitive to microscopic pathophysiology, probing tissue injury at a phase when the distinction between salvageable and irreversibly injured tissue mattered most. The hyperintense lesion on diffusion-weighted images and the corresponding ADC reduction do not indicate simply “more water” or “less water”; they reflect altered water mobility in metabolically challenged tissue (Fig. 2). Although the exact mechanisms remained debated, the clinical impact was immediate: diffusion MRI became the imaging signature of early infarction (Chien et al., 1992; Warach et al., 1992), demonstrating the translational value of preclinical MRI. Subsequent integration with perfusion imaging, vascular imaging, and clinical triage became central to modern stroke workflows, but diffusion imaging supplied the foundational proof of concept.

Stroke also sharpened a methodological issue that remains central today: diffusion MRI is highly sensitive but only moderately specific in isolation. A reduced ADC in acute infarction usually reflects cytotoxic edema and associated microstructural alterations, yet restricted diffusion-like appearances can also occur in abscesses, hypercellular tumors, seizures, toxic-metabolic states, and some encephalopathies. Conversely, ischemic lesions evolve over time; ADC may pseudo-normalize and then rise in subacute stages. Thus, the brain taught diffusion MRI both its greatest triumph and one of its key lessons: sequence contrast must always be interpreted in temporal and clinical context.

The biological meaning of the ADC drop in ischemia has been a subject of intense investigation. The dominant explanatory framework links the signal change to failure of ionic homeostasis, cell swelling, axonal beading, shrinkage of extracellular space, altered membrane-water exchange, and a greater influence of restricted or hindered intracellular motion (Budde & Frank, 2010; Le Bihan & Johansen-Berg, 2012; Moseley, Cohen, Mintorovitch, et al., 1990; Mintorovitch et al., 1994; van der Toorn et al., 1996). Yet no single microstructural mechanism explains all observations across time and tissue compartments. What matters technically is that the ischemic brain gave diffusion MRI a uniquely convincing demonstration that a physical signal sensitive to water motion could carry immediate clinical meaning. Stroke was the bridge between elegant pulse-sequence physics and indispensable medical practice.

Even decades later, acute stroke remains the clearest example of why diffusion MRI should be understood as a dynamic microstructure imaging method rather than merely a specialized contrast weighting. Diffusion MRI allows lesions to emerge before overt structural destruction is visible, and it changes as tissue evolves. In that respect, it occupies a conceptual space between anatomy and physiology. The sequence is not directly imaging metabolism, blood flow, or membrane pumps, but it is exquisitely sensitive to their downstream consequences for microscopic water motion. No other organ provided such an early, dramatic, and clinically useful vindication of the diffusion MRI paradigm (Le Bihan, 2024a).

## 5 White-Matter Anisotropy, Diffusion Tensor Imaging, and the Emergence of Brain Tractography

### 5.1 Diffusion tensor imaging (DTI)

**Fig. 4.**
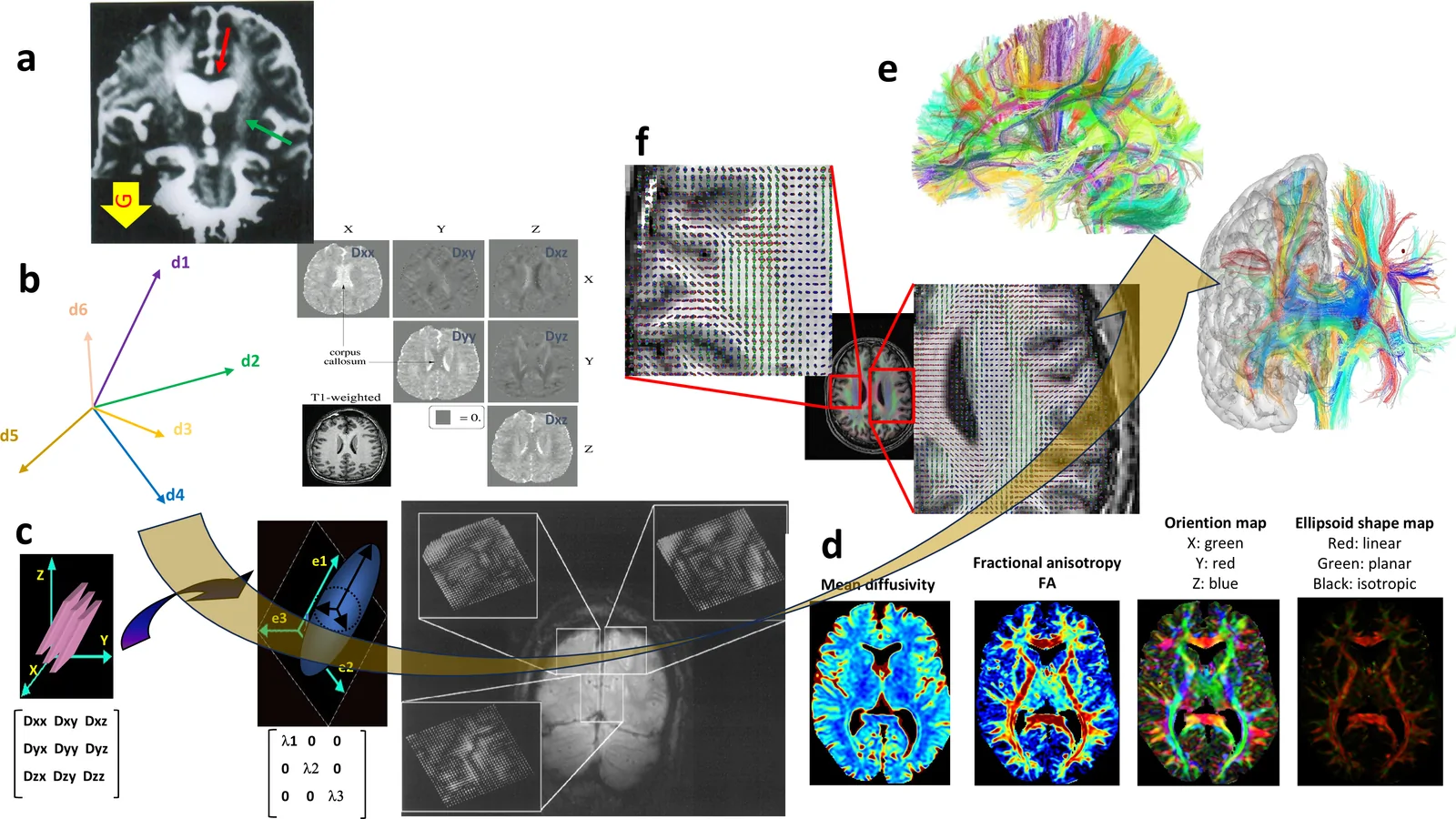
Diffusion tensor imaging. (a) Diffusion is highly anisotropic in the brain, especially in white matter. In this sagittal ADC slice obtained with diffusion encoding along the vertical axis (yellow arrow), the horizontal part of the corpus callosum (red arrow) appears dark, while the vertical corticospinal tract (green arrow) appears bright. (b) To capture full diffusion information on anisotropic tissues, acquisitions must be made with diffusion-sensitizing gradients along at least six noncolinear directions. From this set of six acquisitions, the six independent components of the diffusion tensor in the reference (MRI scanner) space (axes [X, Y, Z]) can be estimated. (c) The “diagonalization” of the diffusion tensor allows to obtain the reference frame (axes [e1, e2, e3]) within which the diffusion tensor becomes diagonal (no off-diagonal term) on a voxel-by-voxel basis. The components λ1, λ2, and λ3 then correspond to the diffusivity along those three axes, which can be represented as a 3D ellipsoid (image copy of the author’s notebook, 1992). (d) From the diffusion tensor and ellipsoid maps, one may derive: Mean diffusivity (directionally averaged diffusion, equivalent to the isotropic ADC), fractional anisotropy (normalized degree of anisotropy), orientation map (taking for the orientation the axis with the highest diffusion, e1), and the ellipsoid shape (linear/cigar shape when λ1 > λ2,3, planar/disk when λ2,3 > λ1 often where fibers cross each other). (e) From the orientation obtained in each voxel, one may draw lines connecting voxels (using arbitrary colors), representing anatomical connectivity (tractography). (f) The Q-ball algorithm allows to sort out voxels where fibers may be crossing, allowing multiple tracts to cross.

If stroke established the clinical value of brain diffusion MRI, white-matter anisotropy established its neuroscientific reach. Long before modern tensor imaging, NMR studies had suggested that water mobility in nervous tissue was direction dependent (Hansen, 1971), but in vivo MRI made this anisotropy visually and quantitatively compelling (Chenevert et al., 1990; Moseley, Cohen & Kucharczyk, 1990; Turner et al., 1990). The central observation was simple: in coherent fiber bundles, diffusion is greater parallel than perpendicular to the bundle. Such anisotropy reflects the ordered architecture of axons and their surrounding microenvironment, even though the MRI voxel is orders of magnitude larger than a single fiber (Beaulieu, 2002). Soon after anisotropy was recognized, color-coded orientation maps of white-matter tracts were produced from diffusion images acquired along different directions, assuming that fibers align with the direction of greatest diffusion (Douek et al., 1991). However, the approach was crude, highlighting the need for a mathematical framework that could represent diffusion in three dimensions: diffusion tensor imaging (DTI) (Fig. 4) (Basser et al., 1994a, 1994b). The diffusion tensor is a second-order symmetric tensor that characterizes, to a first approximation, the local covariance of molecular displacements. Diagonalization yields eigenvalues and eigenvectors, from which mean diffusivity, fractional anisotropy, and principal diffusion directions can be derived. Pierpaoli and colleagues subsequently demonstrated diffusion-tensor MR imaging of the human brain, confirming that tensor-derived metrics and orientations could be obtained in vivo (Pierpaoli et al., 1996).

Formally, the diffusion tensor D is a symmetric 3 × 3 matrix. For a unit encoding direction gˆ and scalar b-value, the signal model reduces to S(b)/S(0) = exp(−b gˆ^ T^Dgˆ). Diagonalization of D yields eigenvalues λ_1_, λ_2_, and λ_3_ and orthonormal eigenvectors that define the principal axes of the diffusion ellipsoid (Basser et al., 1994a, 1994b).

$$\text{MD}=\left(\lambda_{1}+\lambda_{2}+\lambda_{3}\right)/3$$ (6)

$$FA=\sqrt{\left(3/2\right)}\cdot \sqrt{}[({\left(\lambda_{1}-MD\right)}^{2}+{\left(\lambda_{2}-MD\right)}^{2}+{\left(\lambda_{3}-MD\right)}^{2})/(\lambda_{1}^{\;2}+\lambda_{2}^{\;2}+\lambda_{2}^{\;3})].$$ (7)

Mean diffusivity, MD, reports rotationally invariant average mobility, whereas fractional anisotropy, FA, reports the variance of diffusivities relative to their magnitude. Similarly, axial diffusivity λ_1_ and radial diffusivity (λ_2_ + λ_3_)/2 are often discussed mechanistically, but in vivo they remain composite observables rather than unique markers of axonal integrity or myelin content. Noise floor effects, Gibbs ringing, eddy currents, susceptibility distortions, and free-water partial volume can all bias tensor estimates unless explicitly controlled (Beaulieu, 2002; Le Bihan et al., 2001; Tournier et al., 2011).

The introduction of the DTI framework in the brain was a major advance. It converted the qualitative observation of anisotropy into a set of quantitative descriptors. Mean diffusivity provided a rotationally invariant summary of overall water mobility. Fractional anisotropy described the eccentricity of the diffusion ellipsoid and rapidly became the most widely used scalar metric of white-matter organization. The principal eigenvector offered a first estimate of local fiber orientation. The brain’s organized white matter made DTI especially attractive because the tensor ellipsoid could often be interpreted anatomically. Regions such as the corpus callosum, corticospinal tract, and optic radiations exhibited high anisotropy and coherent orientations consistent with known neuroanatomy. For neuroscientists and clinicians alike, DTI offered the first noninvasive whole-brain maps of major white-matter architecture in living humans, turning diffusion MRI from a lesion-sensitive sequence into a platform for investigating normal anatomy, development, aging, and white-matter diseases (see below).

Yet the diffusion tensor also imposed a simplifying assumption: a single Gaussian displacement distribution characterized by a second-order tensor within each voxel. This works surprisingly well in many large white-matter bundles, but it fails in regions with crossing, kissing, branching, or fanning fibers. It also reduces the intricate displacement distribution within tissue to a single ellipsoid. As a result, FA and related metrics are sensitive but not uniquely interpretable. High anisotropy may reflect coherence and packing, whereas low anisotropy may indicate disruption, complex geometry, or the coexistence of multiple orientations within one voxel (Beaulieu, 2002; Pierpaoli et al., 1996; Tournier et al., 2011). The tensor was revolutionary because it made anisotropy measurable, not because it solved the problem of biological specificity.

Despite those limitations, DTI became the common language of brain diffusion MRI. It supported studies of white-matter maturation in infancy and childhood, age-related decline, demyelinating disease, traumatic injury, psychiatric disorders, and the structural correlates of cognition. The reason is pragmatic as well as conceptual: tensor acquisition is relatively efficient, robust, and standardized compared with many advanced methods. Even where more sophisticated models are available, DTI often remains the first analytic layer because it provides a stable orientation field and a familiar set of summary indices (Le Bihan et al., 2001).

The emergence of DTI also changed how the brain was imagined. Before diffusion MRI, whole-brain white-matter anatomy in living humans was inferred indirectly or reconstructed from postmortem dissection. DTI suggested that macroscopic structural connectivity could be charted in vivo, even if only approximately (Assaf et al., 2019; Hagmann et al., 2008). That idea led directly to tractography, and, later, to the modern discourse of the *brain connectome*.

### 5.2 Tractography and the structural connectome

Tractography extended the logic of DTI from local orientation estimation to long-range pathway reconstruction. If each voxel contains an estimate of the dominant diffusion direction, streamlines can be propagated through adjacent voxels to infer the probable trajectories of major white-matter bundles. The first demonstrations (Basser et al., 2000; Conturo et al., 1999; Jones et al., 1999; Mori et al., 1999; Poupon et al., 2000) immediately captured the imagination of the field because they offered something previously unattainable in living humans: a three-dimensional, noninvasive picture of large-scale anatomical connections (Fig. 4).

In the brain, this was far more than a visualization advance. It changed the kinds of questions that could be asked. Structural pathways could now be compared across individuals, developmental stages, disease states, and cognitive phenotypes. Major tracts could be segmented for surgical planning or lesion analysis. Studies began to ask whether disorders previously framed in terms of cortical regions might instead be better understood as network-level disconnections. The language of “white-matter integrity”, “connectivity”, and *brain connectome* became embedded in contemporary neuroscience in large part because tractography made such concepts empirically tractable (Gong, et al., 2009; Hagmann et al., 2003; Koch et al., 2002).

However, tractography is inferential, not photographic. Streamlines are not axons; they are algorithmic reconstructions based on local orientation fields, propagation rules, and stopping criteria. In coherent bundles, the correspondence may be good at a coarse scale, but in regions of crossing or fanning fibers, low anisotropy, edema, tumor infiltration, or severe partial volume, errors can be substantial. The tensor model assumes that diffusion within each voxel can be described by a single dominant orientation, an assumption that breaks down when multiple fiber populations coexist within a voxel. In such regions, tensor-derived metrics may be ambiguous or misleading. False positives and false negatives are both common: pathways may terminate spuriously, traverse implausible routes, or miss genuine but sharply curving projections. Tractography, therefore, visualizes a model of anatomical connectivity constrained by diffusion data, not anatomy itself (Daducci et al., 2016; Tournier et al., 2011).

These problems were already implicit in DTI-based streamline methods and became more obvious as researchers attempted whole-brain connectomics. The promise was irresistible: if one could parcellate the cortex and estimate the streamlines linking each pair of regions, one could generate a graph of the human brain’s structural network and relate it to functional MRI, behavior, development, and disease. Diffusion MRI became a foundational tool for the Human Connectome era because it offered a scalable route to individual structural connectivity (Assaf et al., 2019; Hagmann et al., 2003). At the same time, the field learned that graph metrics are only as meaningful as the tractography from which they are derived. Small changes in acquisition, reconstruction, or filtering can alter connectivity matrices and downstream inferences.

To mitigate the single-tensor crossing-fiber limitation, high-angular-resolution diffusion imaging (HARDI) acquires diffusion-weighted data along many more directions than the minimum six required for tensor estimation (Frank, 2002). Methods such as Q-ball imaging (Tuch, 2004) and diffusion spectrum imaging (DSI) (Wedeen et al., 2005) use richer q-space sampling to estimate an orientation distribution function (ODF), representing the relative prominence of water diffusion along different spatial directions within a voxel. Multiple ODF peaks may, therefore, reveal distinct fiber populations in regions of crossing, kissing, or fanning fibers. High diffusion weighting can increase sensitivity to restricted, slowly diffusing components, including a relative intra-axonal contribution, suppressing a lot of extracellular water signal, which could make a clearer identification of principal diffusion directions. However, these approaches remain sensitive to motion, distortion, partial-volume effects, and reconstruction choices, and have reduced signal-to-noise ratio at high b-values, though this low SNR can be mitigated by the large number of directions acquired (at a cost of longer acquisitions). Importantly, ODF peaks reflect preferential diffusion orientations rather than individual axons or proven anatomical pathways; tractography based on them may still produce false-positive or false-negative connections, particularly in regions of complex geometry or low angular separation between fiber populations. Probabilistic approaches have also been used to create the first atlases of large-scale brain connections (Behrens et al., 2003; Hagmann et al., 2003; Parker et al., 2003). These innovations substantially improved the representation of orientation complexity in the brain and expanded the domains in which tractography could operate credibly.

Even so, the conceptual caution remains the same: tractography works best as a mesoscale anatomical inference tool. It is highly valuable for identifying major pathway organization, asymmetries, developmental changes, and lesion-induced disconnection patterns. It is less secure as a direct axon-counting technology or as a precise map of synaptic communication. The field increasingly recognizes that diffusion MRI constrains connectivity rather than measuring it exhaustively. Structural connectome analyses are most convincing when integrated with neuroanatomical knowledge, functional data, and careful control of tractography biases (Assaf et al., 2019; Daducci et al., 2016).

Nonetheless, the impact of tractography on brain science has been enormous. It has reoriented neurosurgical planning (Yang et al., 2021), enabled in vivo studies of language and motor pathways, and provided a framework for rethinking neurological and psychiatric disorders as disorders of networks (Berkovitch et al., 2021). The shift from localized lesions to distributed circuits owes much to the way diffusion MRI made brain wiring visible, albeit imperfectly. In that sense, tractography is one of the defining achievements of the brain-centered diffusion MRI story (Assaf et al., 2019; Gong, et al., 2009).

## 6 What Diffusion MRI Measures in Brain Tissue: Non-Gaussian Behavior and Brain Microstructure Models

### 6.1 The diffusion landscape

First, it should be clarified that this article considers only water diffusion. Diffusion MRI has also been used to investigate the diffusion of nonwater molecules in the human brain, notably metabolites and neurotransmitters (Posse et al., 1993).

Biological tissues are dynamic, hierarchically organized systems whose molecular composition and cellular architecture shape not only mechanical and electrical properties (see below), but also water mobility. Water interacts continuously with proteins, lipids, membranes, and organelles, while macromolecular crowding, membrane permeability, cell packing, extracellular geometry, and tissue organization all influence diffusion, making diffusion MRI uniquely suited to provide a window into tissue microarchitecture. It can detect tissue features at the scale where cellular organization (and pathology) emerge, without directly imaging them.

Membranes constrain water motion by separating intracellular and extracellular spaces. Restricted diffusion refers to confinement by boundaries within the diffusion time, whereas hindered diffusion describes slowed but unconfined motion through complex, tortuous environments (Nicholson & Phillips, 1981; Nicholson & Syková, 1998; Nilsson et al., 2013). Directionally organized structures, such as aligned axons or tissue layers, produce anisotropic diffusion (Beaulieu, 2002). These mechanisms coexist within each voxel, generating non-Gaussian and time-dependent diffusion behavior (Fieremans et al., 2016; Novikov et al., 2019).

Pathological processes—including edema, inflammation, tumor infiltration, fibrosis, necrosis, and treatment effects—modify tissue microarchitecture and, therefore, diffusion signals. Reduced diffusion may indicate hypercellularity but is not specific. Diffusion MRI does not directly resolve microscopic structures; rather, it measures their ensemble effect on water motion, providing microstructural sensitivity beyond its nominal spatial resolution.

### 6.2 Modeling diffusion in tissues

#### 6.2.1 “Shut-up and calculate” empirical/phenomenological models

As gradient hardware improved and acquisitions extended to higher b-values and multiple diffusion times, it became increasingly clear that a mono-exponential model, such as Eq. (3) and the DTI framework, captures only a subset of the information present in brain diffusion data. Signal decay in both white and gray matter often deviates substantially from the Gaussian assumption. These deviations are not merely fitting residuals; they encode the interaction of water with complex tissue geometry, exchange, and multiscale organization. Diffusion kurtosis imaging (DKI) was among the earliest MRI approaches developed to describe empirically the curvature of the diffusion-weighted MRI signal decay with b-value, reflecting the underlying non-Gaussian displacement probability distribution (Jensen et al., 2005). A mathematically driven approach rewrites the diffusion term as a polynomial expansion in *b*. The kurtosis K is associated with the second-order term and quantifies deviation from a Gaussian distribution, which is linear in *b*:

$$\text{S}\left(\text{b}\right)/\text{S}\left(0\right)=\text{exp }\left[-{\text{bADC}}_{0}+{\left({\text{bADC}}_{0}\right)}^{2}\text{K}/6\right],$$ (8a)

which, to be complete, can be merged with the IVIM effects visible at low b values:

$$\begin{array}{l} \text{S}\left(\text{b}\right)/\text{S}\left(0\right)=\{{\text{f}}_{\text{IVIM}}\text{ exp}\left(-{\text{bD}}^{*}\right)+\left(1-{\text{ f}}_{\text{IVIM}}\right)\\ \;\;\;\;\;\;\;\;\;\;\;\;\;\;\;\;\;\;\;\;\;\text{exp }\left[-{\text{bADC}}_{0}+{\left({\text{bADC}}_{0}\right)}^{2}\text{K}/6\right]\}, \end{array}$$ (8b)

where ADC_0_ is the extrapolated ADC when *b* approaches zero (Fig. 3). Importantly, because this model is a second-order expansion on *b*, it breaks down at very high b-values (e.g., around 3,000 s/mm² for typical ADCo and K values found in tissues), requiring higher-order terms. In anisotropic tissues, especially white matter, kurtosis is itself direction dependent and cannot be fully characterized by a single Mean Kurtosis value, but using a fourth-order tensor. Diffusion kurtosis imaging can, therefore, provide directional metrics such as axial kurtosis and radial kurtosis, and more specifically diffusional kurtosis anisotropy, which quantify how non-Gaussian diffusion varies with fiber orientation and may reveal microstructural features not captured by conventional diffusion anisotropy alone (Glenn et al., 2015).

In biological tissues, increased kurtosis is generally interpreted as reflecting greater microstructural complexity, restriction, or hindrance. DKI has proved sensitive to pathological changes in ischemia, neurodegeneration, and tumors (Helpern & Jensen, 2015; Iima & Le Bihan, 2016). A relationship between mean kurtosis and cortical myelin content has been reported in primates (Reveley et al., 2024); however, kurtosis parameters remain descriptive rather than explanatory because they quantify non-Gaussianity without identifying its specific biological origin. Interpretation, therefore, requires caution. Other empirical approaches include the stretched-exponential model (Yablonskiy & Sukstanskii, 2010; Yablonskiy et al., 2003).

#### 6.2.2 Biophysical models

This identifiability problem is central. Brain tissue is highly complex, whereas the MR signal is finite, indirect, and noisy. Early approaches attempted to model curvature in diffusion signal decay arising from co-existence of multiple compartments (Stanisz et al., 1997), sometimes without assigning each component to a specific microstructural entity (Le Bihan, 1995, 2007; Niendorf et al., 1996). More recent biophysical diffusion models may be viewed as attempts to factor the signal further into compartments, statistical moments, or tissue-specific features while preserving identifiability. In its simplest form, the normalized signal can be written as a weighted sum over k compartments:

$$\text{S}\left(\text{b},\text{ Tdiff},\hat{g}\right)/\text{S}\left(0\right)=\sum {\text{f}}_{\text{k}}{\text{S}}_{\text{k}}\left(\text{b}\right),\text{ Tdiff},\hat{g},\theta_{\text{k}}),\text{ with }\sum {\text{f}}_{\text{k}}=1,$$ (9)

where f_k_ are signal fractions and θ_k_ denote compartment-specific parameters such as diffusivities, orientation dispersion, axon diameter, soma size, or exchange rates. Time dependence provides an additional dimension of contrast, because barriers, disorder, and exchange are probed differently as diffusion length changes. Diffusion MRI is, therefore, most informative when acquisitions are diversified across b-values, gradient directions, diffusion times, and encoding waveforms, rather than reduced to a single shell or single summary metric.

In white matter, what is now often called the “Standard Model” of diffusion MRI provides a useful organizing framework. It represents the voxel signal as the sum of intra-axonal, extra-axonal, and sometimes isotropic free-water-like contributions, integrated over the fiber orientation distribution. The intra-axonal compartment is usually modeled as impermeable, zero-radius cylinders, or “sticks” (as opposed to cylinders) with diffusion occurring mainly along the axonal axis and negligible radial diffusivity (McKinnon et al., 2017; Veraart et al., 2019). The extra-axonal space is modeled as an anisotropic Gaussian compartment, often described by an axially symmetric diffusion tensor. A third isotropic compartment may be added to account for CSF or free water. This framework underlies, or is closely related to, several influential models and approaches, including CHARMED, AxCaliber, NODDI, WMTI, SMT, and related multi-compartment formulations (Assaf & Basser, 2005; Assaf et al., 2008; Zhang et al., 2012). Those assumptions make the model tractable, but also fragile. A major limitation is that parameter estimation is often ill-conditioned: different combinations of intra-axonal fraction, extra-axonal diffusivities, orientation dispersion, free water, exchange, and noise can produce very similar signals. This degeneracy is particularly problematic when data are limited in b-value range, angular coverage, diffusion time, gradient waveform diversity, or signal-to-noise ratio (Jelescu & Budde, 2017; Novikov et al., 2019). In other words, the data may not uniquely support the biological interpretation one wishes to make, though subsequent theoretical work has characterized this degeneracy and shown that the model parameters can be distinguished with sufficiently informative acquisition protocols or additional measurements (Coelho et al., 2019; Reisert et al., 2019).

White-matter studies have nevertheless demonstrated the value of this approach. CHARMED and AxCaliber were influential early attempts to estimate restricted fractions and axon caliber distributions from diffusion data (Assaf & Basser, 2005; Assaf et al., 2008). Later work showed that human white-matter diffusivity depends on diffusion time, supporting the idea that axonal packing, extra-axonal disorder, restrictions, and exchange contribute to the measured signal (Fieremans et al., 2016). These observations reinforce the broader view that diffusion MRI can provide a mesoscopic assay of tissue geometry, provided that model complexity is matched to the acquisition protocol and that the resulting parameters are interpreted as model-dependent estimates rather than direct histological measurements (Jelescu & Budde, 2017; Jelescu et al., 2016; Novikov et al., 2019).

Gray matter presents a different and arguably greater challenge. The cortical ribbon lacks the overt orientational coherence of large white-matter bundles, but it contains highly structured arrangements of dendrites, axons, somata, glial processes, capillaries, and extracellular spaces. Historically, diffusion MRI in gray matter was underexploited because tensor metrics are less visually striking than in white matter and because partial-volume contamination from CSF is substantial. The white-matter Standard Model is, therefore, increasingly viewed as insufficient for gray matter, where soma signal contribution, dendritic geometry, inter-compartment exchange, and non-Gaussian diffusion along neuronal and glial processes may need to be included. Zhang et al. (2012) introduced clinically feasible indices of neurite density and orientation dispersion (NODDI). SANDI and related SANDIX approaches extended this logic by explicitly incorporating soma and neurite contributions (Olesen et al., 2022; Palombo et al., 2020). NEXI/SMEX address gray matter more directly by including inter-compartment exchange in a minimal model tailored to such tissue (Jelescu et al., 2022; Olesen et al., 2022). These approaches similarly reflect the need to move beyond white-matter assumptions when modeling cortical and deep gray-matter microstructure.

The present landscape is, therefore, nuanced. Advanced diffusion MRI methods clearly contain microstructural information beyond conventional DWI and DTI, and they are likely to be important for the next generation of brain studies. Yet “biophysical parameter” does not automatically mean “direct biological measurement.” Model outputs remain dependent on the acquisition protocol, mathematical constraints, prior assumptions, and noise properties. This does not make such models unhelpful; on the contrary, they have advanced the field by forcing explicit hypotheses about which aspects of tissue organization are measurable. It does mean, however, that interpretive ambition must be balanced by methodological restraint, especially in the brain where anatomy, pathology, development, aging, and function interact across multiple spatial and temporal scales (Assaf et al., 2019; Novikov et al., 2019).

From a practical standpoint, the most fruitful strategy is often hierarchical rather than competitive. Conventional DWI and DTI provide robust sensitivity, lesion conspicuity, ADC or diffusivity metrics, and orientation fields. Higher-order approaches then address more specific questions in targeted settings: Does kurtosis reflect tissue heterogeneity; does free-water correction sharpen pathological contrast; does diffusion-time dependence reveal a characteristic length scale; does orientation dispersion explain apparent diffusivity changes; does an exchange model better account for cortical diffusion? Such a layered workflow is particularly appropriate for the brain, where no single metric or model can capture the full biological complexity of tissue structure and function (Novikov et al., 2019; Tournier et al., 2011).

#### 6.2.3 sADC and S-index

From a clinical standpoint, an important limitation of empirical diffusion models, and even more so of biophysical models, is their need for extensive datasets spanning multiple b-values, gradient directions, and diffusion times. Such requirements significantly increase acquisition time, which remains a major constraint in clinical MRI. We introduce here two recently introduced body diffusion MRI markers that respond to this demand, although their potential has not yet been established for the brain.

A simple way to enhance sensitivity to non-Gaussian diffusion while preserving clinical feasibility is the shifted ADC (sADC) (Iima & Le Bihan, 2016). Because non-Gaussian diffusion effects become more prominent at higher b-values, whereas perfusion-related IVIM effects are mainly confined to very low b-values, the standard ADC can be replaced by an sADC calculated from two “key” b-values. In body imaging, these are typically around 200 and 1,500 s/mm²; in the brain, the higher key b-value is slightly greater, around 1,800 s/mm². The sADC is defined as:

sADC= ln[S(Lb)/S(Hb)]/(Hb−Lb). (10)

This approach retains the short acquisition time of standard ADC while improving sensitivity to tissue microstructure. It has proved useful for differentiating tissue types and classifying benign and malignant breast and prostate lesions, with reported correlations with histological biomarkers (Goto et al., 2019, 2022, 2025; Wibulpolprasert et al., 2026). The potential of this marker to classify brain tissues and lesions, however, remains to be established. A first step would be to apply the DTI framework using the two key b-values (in which case the equivalent of sADC would be a shifted mean diffusivity, sMD).

**Fig. 5.**
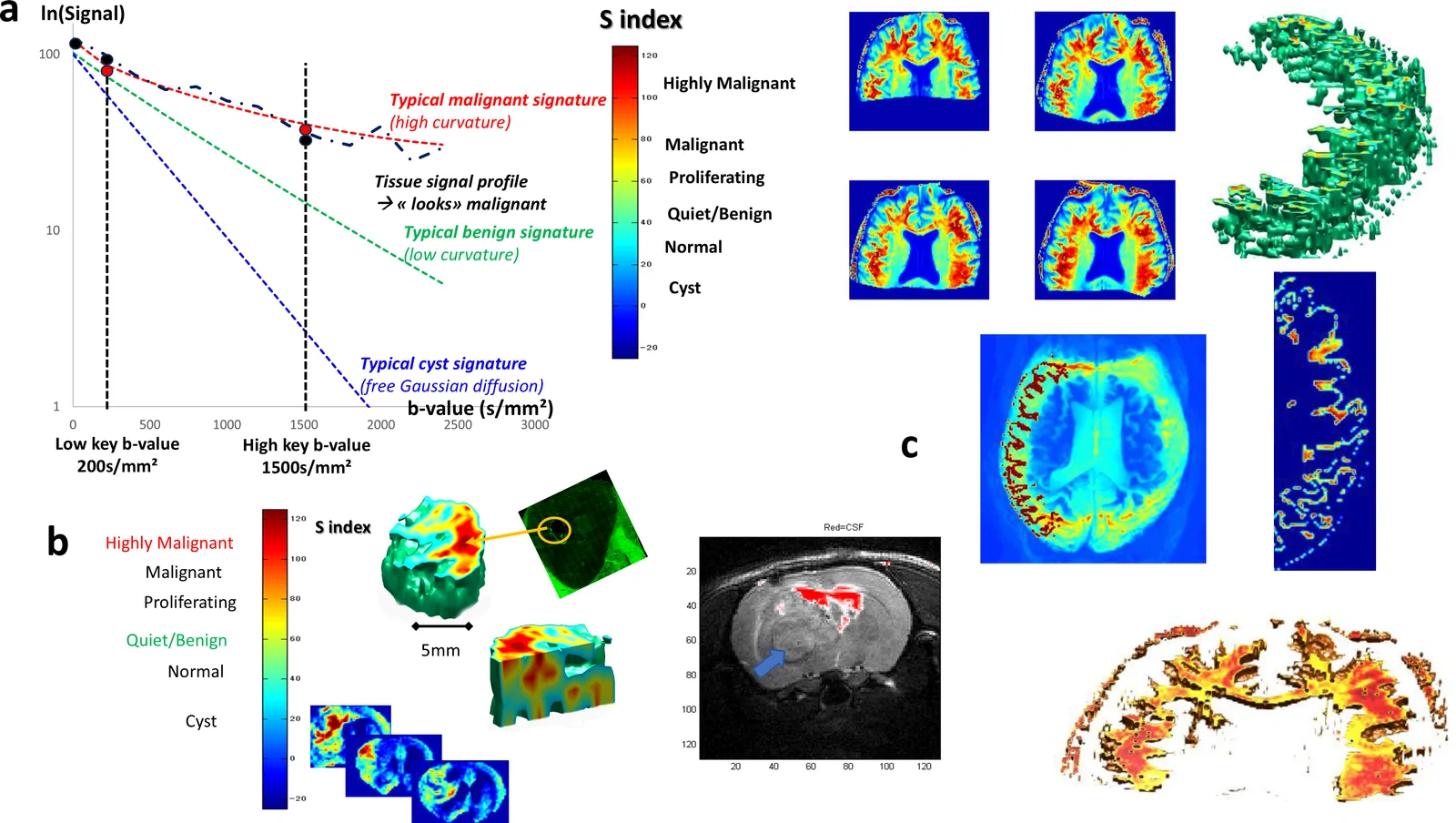
S-index. (a) The S-index is calculated from the proximity of the profile of the diffusion-weighted signal under investigation (dark dotted line) with previously established signal profiles of signature tissues (here a typical malignant lesion, red, and a typical benign lesion, green) at key b-values (for instance 200 and 1,500 s/mm²). The S-index scale allows lesions to be automatically classified according to their S-index value. (b) Example of S-index 3D maps in a rat brain 9L glioma model with corresponding S-index scale. Adapted, in part, with permission of the Radiological Society of North America (RSNA), from Iima and Le Bihan (2016). (c) Examples of 3D S-index maps obtained with key b-values of 200 and 1,800 s/mm² in the normal brain obtained at 7T highlighting differences in gray and white matter related to underlying microstructure otherwise difficult to visualize using standard DTI. (Collaboration T. Yamamoto, M. Fukunaga and N. Sadato, National Institutes for Physiological Studies, Okazaki, Japan).

A further conceptual step is to move beyond explicit model fitting altogether and classify tissues directly from their diffusion signal patterns. One example is the Signature Index (SI), which compares measured diffusion signal profiles with a library of predefined reference signatures derived from training cohorts, rather than estimating parameters such as ADC or kurtosis (Iima & Le Bihan, 2016). These signatures may be derived from models or learned through artificial-intelligence approaches. For each voxel or region of interest, the normalized signal is compared with reference profiles, and a similarity measure, the SI, is computed (Fig. 5). This compresses information across b-values into a single scalar reflecting how closely tissue matches a given category, such as gray matter or white matter, tumor, cyst, inflammation, or necrosis, as initially demonstrated in a preclinical brain-tumor model (Iima & Le Bihan, 2016). Because only two optimized b-values may suffice and no fitting is required, the method is simple and computationally efficient. More broadly, this signature analysis shifts diffusion MRI from parameter estimation toward direct pattern classification, with strong potential for rapid, contrast-free tissue characterization in clinical and research settings.

A key message for clinical efficiency is that accurate classification may be achievable using only a limited number of b-values. SI computation requires no parameter fitting and can permit near real-time analysis. Clinical studies have demonstrated diagnostic performance for breast and prostate cancers comparable with, or exceeding, that of advanced diffusion models, with strong correlations to histopathological biomarkers (Goto et al., 2019, 2022, 2025; Wibulpolprasert et al., 2026). Overall, signature-based diffusion MRI represents a shift from *parameter estimation* to *pattern recognition*, avoiding complex modeling while preserving biologically meaningful contrast, thus offering a practical, model-free approach to quantitative tissue characterization. Here also, however, the potential of this Signature Index to classify brain tissues and diseases remains to be established. A straightforward approach consists in deriving the Signature Index from geometrically averaged signals acquired at key b-values (Fig. 5c), but the Signature Index could also benefit from integrating diffusion anisotropy features by using libraries of directional signal signatures.

## 7 Major Neurological Applications Beyond Acute Stroke

After its rapid clinical adoption in acute ischemic stroke and the subsequent development of tractography, diffusion MRI became a central tool for investigating brain disorders and development. Beyond the cerebral cortex, diffusion MRI provides a sensitive window on deep gray-matter nuclei, including the thalamus, basal ganglia, hippocampus, amygdala, and brainstem nuclei. In these structures, diffusion is usually less directionally organized than in white matter; consequently, mean diffusivity, kurtosis, free-water, and multi-shell microstructural indices are often more informative than fractional anisotropy alone. Such measures can reveal changes related to neuronal and dendritic loss, gliosis, extracellular-water expansion, iron-associated tissue alterations, or disruption of intrinsic nuclear organization in aging, neurodegeneration, inflammatory disease, and movement disorders. In white-matter disease broadly construed, DTI provides sensitive markers of altered water mobility and orientational coherence that may arise from demyelination, axonal injury or degeneration, edema, gliosis, and fiber disorganization. These measures are not, however, specific to any single pathological process and must be interpreted in light of tissue geometry, partial-volume effects, acquisition conditions, and the underlying diffusion model (Jelescu & Budde, 2017). The appeal is evident: many neurological and psychiatric disorders affect distributed pathways rather than isolated lesions, and diffusion MRI offers a whole-brain, noninvasive framework for studying microstructure and anatomical connectivity.

Inflammatory and demyelinating disorders, traumatic brain injury (TBI), epilepsy, and neuro-oncology illustrate both the breadth and limitations of diffusion MRI. In multiple sclerosis, abnormal diffusion extends beyond focal plaques into normal-appearing white and gray matter, supporting a diffuse disorder of tissue integrity and network architecture. Free-water, neurite, kurtosis, and diffusion-basis-spectrum methods may increase sensitivity to extracellular water and axonal injury, but their pathological specificity remains insufficient to replace conventional lesion, atrophy, or susceptibility imaging (Caranova et al., 2023; Jalilian et al., 2024). In TBI, DWI can detect nonhemorrhagic axonal injury, while DTI and DKI demonstrate group-level abnormalities in the corpus callosum and association tracts. Yet these parameters are nonspecific and cannot confirm or exclude traumatic axonal injury in an individual with concussion (Tsiouris & Lui, 2024). In drug-resistant focal epilepsy, diffusion abnormalities and tractography support a network view of disease and can inform risk assessment near visual, motor, and language pathways (Fiallo Arroyo & Leon-Rojas, 2025; Kamagata et al., 2024).

Developmental neuroscience has been one of the major beneficiaries. From late gestation through infancy, childhood, and adolescence, mean diffusivity generally declines while fractional anisotropy rises, reflecting the combined effects of changing tissue water, axonal organization and packing, membrane barriers, fiber coherence, and myelination. These metrics should not be interpreted as direct measures of myelin or axonal density, but they have enabled in vivo mapping of tract-specific maturation and its links with emerging motor, language, and cognitive functions (Dubois et al., 2021; Lebel et al., 2019). Diffusion MRI has also made it possible to identify deviations from typical developmental trajectories in prematurity, cerebral palsy, autism spectrum disorder, and neurogenetic conditions. Wolff and colleagues reported altered tract development between 6 and 24 months in infants subsequently diagnosed with autism, suggesting that atypical structural maturation may appear early, preceding overt behavioral diagnosis (Wolff et al., 2012). Recent reviews nevertheless emphasize marked heterogeneity across ages, tracts, clinical profiles, and analytical approaches; diffusion MRI, therefore, currently informs developmental mechanisms and stratification rather than individual diagnosis (Halliday et al., 2024).

Psychiatry has likewise embraced diffusion MRI under the hypothesis that disorders of cognition and behavior may involve dysconnectivity of distributed systems rather than only focal gray-matter abnormalities. Schizophrenia remains the best-established example: the ENIGMA Schizophrenia DTI Working Group identified widespread, although modest, microstructural differences involving association, commissural, and projection pathways in 4,322 individuals (Kelly et al., 2018). Subsequent free-water meta-analysis supports an additional extracellular-water component, but the biological basis of altered diffusion remains heterogeneous and may reflect different contributions from axons, myelin, extracellular water, inflammation, medication exposure, and illness course (Carreira Figueiredo et al., 2022). More focused work has linked long-distance structural connectivity within the global neuronal workspace to impaired conscious access and psychotic symptoms (Berkovitch et al., 2021). In neurogenetic disorders such as 22q11.2 deletion syndrome, advanced diffusion MRI has revealed reduced tract volumes and microstructural features consistent with atypical axonal morphology, strengthening the developmental and translational network perspective on psychosis vulnerability (Raven et al., 2023).

Brain tumors are an important application of diffusion MRI within multiparametric neuro-oncological imaging. Diffusion-weighted imaging and ADC maps contribute to the assessment of tumor cellularity, grade, necrosis, and peritumoral abnormalities. Restricted diffusion is often associated with hypercellularity, but ADC is also influenced by edema, necrosis, hemorrhage, microvascular architecture, and intratumoral heterogeneity (Falk Delgado, 2025). More advanced diffusion methods, including DKI and multi-shell models, may better capture the mixture of infiltrating tumor, vasogenic edema, and disrupted white matter; in a standardized multicenter study, diffusion kurtosis combined with dynamic contrast-enhanced perfusion MRI improved diagnostic confidence and glioma-subtype prediction (Richter et al., 2024). Diffusion MRI may also provide complementary information for treatment monitoring, but no single diffusion marker can reliably distinguish viable tumor from pseudo-progression or radiation necrosis (Lawrence et al., 2025). Tractography is increasingly used to estimate the relationship between tumors and eloquent pathways before surgery (Kamagata et al., 2024; Yang et al., 2021), but infiltration, edema, mass effect, crossing fibers, reduced anisotropy, algorithmic choices, and intraoperative brain shift may generate false-positive or false-negative reconstructions. It should, therefore, complement, rather than replace, functional mapping and intraoperative stimulation (Koga et al., 2024).

Normal aging and neurodegeneration constitute another major application domain for diffusion MRI. Across adulthood, diffusion measures show tract-specific and nonlinear changes, including increasing mean and radial diffusivity and declining fractional anisotropy in later life. Tract-based analyses have mapped age-related decline and disease-specific pathway vulnerability (Gong et al., 2014; Sullivan & Pfefferbaum, 2006). However, these measures reflect a composite of axonal organization, myelin, extracellular water, fiber geometry, and cerebrovascular injury (Beck et al., 2021; Jones et al., 2013). At symptomatic onset, absolute diffusivity measures may be more sensitive than fractional anisotropy, whereas increasing radial diffusivity and declining fractional anisotropy may better track disease severity longitudinally (Acosta-Cabronero et al., 2010, 2012; Chen et al., 2023; Fu et al., 2014; Lo Buono et al., 2020). In mild cognitive impairment and Alzheimer’s disease, diffusion abnormalities may precede marked macroscopic atrophy and commonly involve limbic and posterior association pathways, including the fornix, cingulum, corpus callosum, and temporoparietal white matter. White-matter free water has shown associations with cognitive performance and subsequent decline, particularly in limbic tracts (Nakaya et al., 2022; Peter et al., 2025). Multi-shell approaches, including NODDI, constrained NODDI (C-NODDI), and diffusion kurtosis imaging, provide model-dependent indices related to neurite organization, extracellular water, axonal density, and tissue integrity (Alsameen et al., 2023; Raghavan et al., 2021; Schilling et al., 2022). Recent longitudinal work further suggests that diffusion-derived axonal-density measures may detect early, spatially selective degeneration related to clinical decline in Alzheimer’s disease (Gong et al., 2026; Wang et al., 2023). These measures are promising candidate biomarkers that complement structural MRI, molecular biomarkers, and clinical assessment.

Diffusion MRI, particularly DTI tractography and connectomic analysis, is increasingly important in Parkinson’s disease because therapeutic efficacy depends not only on reaching a target nucleus, such as the subthalamic nucleus or internal globus pallidus, but also on modulating the appropriate motor and associative networks. Preoperative tractography can guide deep brain stimulation (DBS) or MR-guided focused ultrasound (MRgFUS) planning, identify the structural connectivity of the volume of tissue activated, and help avoid pathways associated with motor, cognitive, or mood-related adverse effects (Pujol et al., 2017; Rajamani et al., 2024; Vanegas-Arroyave et al., 2016). More broadly, diffusion-based network mapping supports the view of Parkinson’s disease as a somato-cognitive action-network disorder (Ren et al., 2026).

Diffusion MRI has also become central to basic neuroscience questions concerning the relationship between structural and functional organization. Studies combining tractography with functional MRI examine how anatomical pathways constrain resting-state and task-evoked networks, how structural and functional connectivity correspond, and where they diverge. Early MRI work established this multimodal framework (Koch et al., 2002), while later connectomic studies extended it to whole-cortex graphs and network topology (Gong, et al., 2009). More recent analyses show that structure–function coupling is regionally heterogeneous, changes across development, and relates to cognitive performance, implying that physical wiring constrains but does not determine brain dynamics (G. Feng et al., 2024; Popp et al., 2025). Diffusion MRI is thus indispensable for a modern account of how large-scale brain systems are physically scaffolded, provided that tractography is interpreted as an indirect, model-based estimate of anatomical connectivity.

The breadth of these applications helps explain why the brain remains the center of gravity for diffusion MRI. Other organs certainly use diffusion imaging, sometimes with enormous clinical success, but no other field has built such a comprehensive ecosystem around it, spanning emergency diagnosis, developmental neuroscience, systems neuroscience, psychiatric network analysis, neurosurgical planning, and debates about direct functional imaging. The brain gave diffusion MRI not only its first clinical triumph but also its richest scientific agenda.

## 8 Emerging Frontiers

This section introduces emerging diffusion-MRI concepts that remain at an early stage of development. These “frontier” approaches should be distinguished from more established advanced methods, such as biophysical diffusion models, which have been developed over many years, even if they have not yet been fully deployed in clinical practice, as presented in the preceding applications section and in other articles of this special issue. By contrast, frontier concepts, such as diffusion-based virtual magnetic resonance elastography (vMRE), are forward-looking approaches with substantial potential, although they remain to fully evaluate. Initial clinical studies in the body and brain provide a foundation for further development. Diffusion functional MRI is likewise attracting attention, particularly as the limitations of conventional neurovascular-based methods such as BOLD fMRI become increasingly evident. As for the relativistic-connectome framework, it is more speculative, advocating that the distribution of action-potential propagation velocities across the connectome may be as important as structural and functional connectivity. Advanced diffusion MRI could, in principle, contribute to estimating the relevant conduction properties and thereby illuminate mechanisms and phenotypic diversity in psychiatric disorders and, potentially, consciousness.

### 8.1 Functional IVIM and diffusion MRI

#### 8.1.1 Early IVIM attempts

In the mid-1980s, one aim in developing IVIM imaging was to produce cerebral-perfusion maps for studying brain function (Fig. 2). This goal was based on the established relationship between neuronal activation, metabolism, and blood flow—namely, neurovascular coupling—which PET was already exploiting successfully (Roy & Sherrington, 1890). IVIM-based fMRI could not compete once alternative methods emerged in the early 1990s. After an initial approach using contrast agents (Belliveau et al., 1991), BOLD (blood-oxygen-level-dependent) fMRI rapidly became dominant (Ogawa et al., 1992) because it was easier to implement and offered greater sensitivity. Consequently, the technically more demanding IVIM approach was largely set aside. Later studies nevertheless confirmed the validity of the IVIM concept: increases in low-b ADC and IVIM perfusion parameters were observed in activated brain regions, also revealing contributions from different vessel types to the fMRI signal (Song et al., 1996). IVIM MRI has also been combined with BOLD fMRI in a complementary “negative” approach that improves spatial specificity by suppressing signals from large feeding arteries and draining veins (Song et al., 2018).

#### 8.1.2 Diffusion fMRI

While BOLD fMRI transformed cognitive neuroscience, it remains an indirect measure of neural activity through neurovascular coupling (Roy & Sherrington, 1890). Because the BOLD response depends on local changes in cerebral blood flow, blood volume, and oxygenation, it does not always track underlying neuronal signaling faithfully and can diverge from activation-driven oxygen metabolism in some regions or conditions (Epp et al., 2026; Logothetis & Wandell, 2004; Sirotin & Das, 2009). It is also vulnerable to pathology, anesthesia, and vasoactive drugs, while vascular anatomy and delayed hemodynamic responses constrain spatial specificity and temporal resolution (Sirotin & Das, 2009; Turner, 2002).

**Fig. 6.**
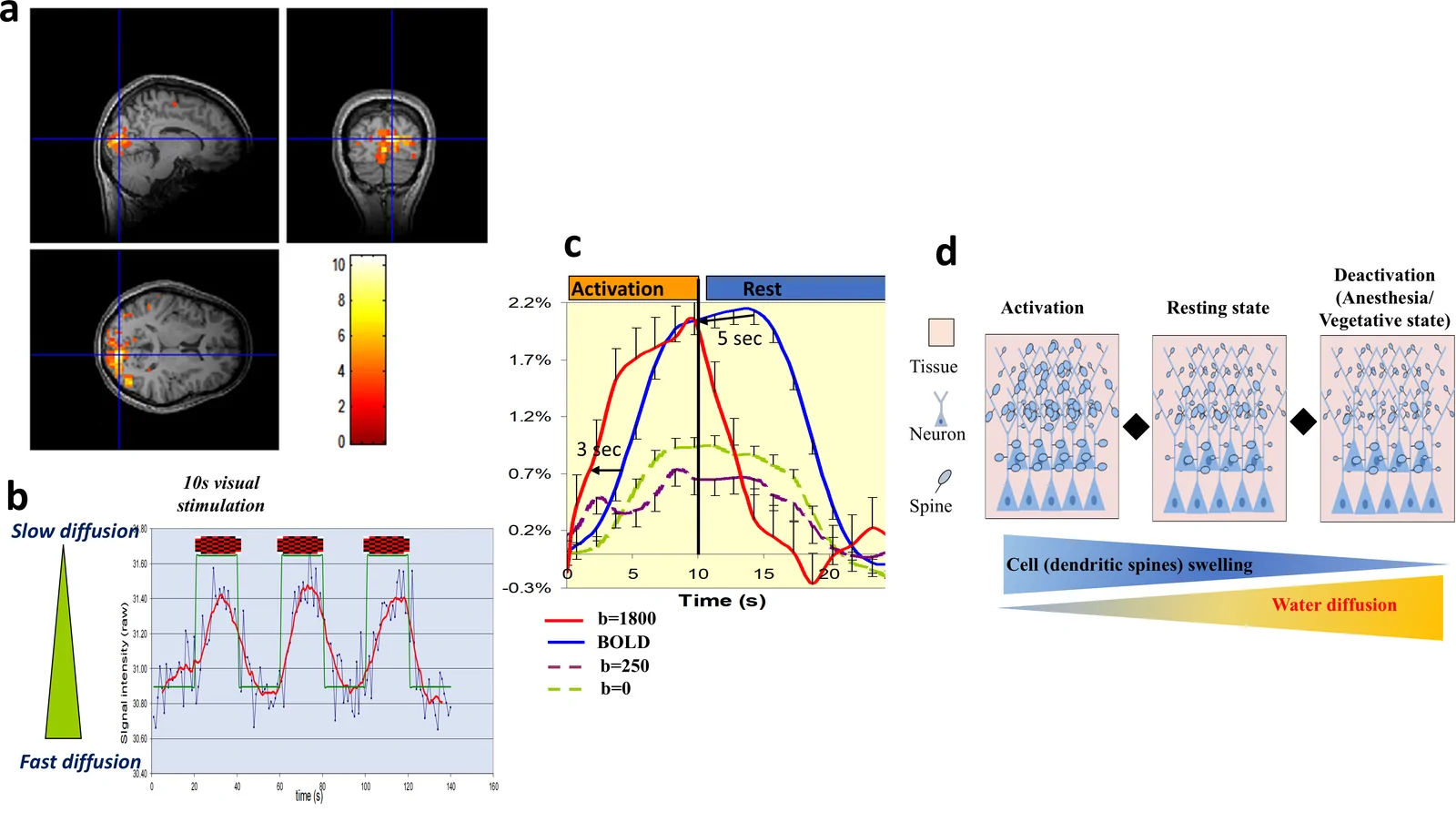
Diffusion functional MRI. (a) Diffusion fMRI activation maps showing visual cortex activation obtained in the visual cortex of a normal volunteer subject to periods of 10-second stimulation by a flickering checkerboard (adapted in part from Le Bihan et al. (2006), originally published in Proceedings of the National Academy of Sciences). (b) During visual stimulation, the diffusion-weighted MRI signal goes up, reflecting a slowdown in water diffusion. (c) The time course of the signal response over the 10-second stimulation window shows that the diffusion response starts and ends faster than the BOLD fMRI response, more closely reflecting the stimulation time course. The response amplitude between BOLD and the diffusion response (with b = 1,800 s/mm²) is similar. Note that a response is visible at low b values (b = 0 and 200 s/mm²) corresponding to a residual contribution of the BOLD signal (T2/T2’ effect). The amplitude at b = 250 mm/s² is slightly lower than at b = 0 s/mm² due to the crushing effect of the diffusion-encoding gradients on flowing blood. The higher response at b = 1,800 s/mm² is a signature of the diffusion-related nature of the response. (d) Putative mechanism involved with DfMRI: Neuronal activation results in the dynamic swelling of a very large number of dendritic spines that hinders water diffusion. At rest, and even more in deactivation produced by anesthesia or vegetative states, the amount of dynamic swelling is lower, resulting in higher diffusion.

A major conceptual shift occurred in the early 2000s when diffusion MRI was shown to detect brain activation through transient decreases in ADC, suggesting sensitivity to intrinsic microstructural changes in addition to possible vascular effects (Fig. 6) (Darquié et al., 2001; Le Bihan et al., 2006). In human visual cortex, these diffusion changes preceded the BOLD response and were closely time locked to stimulation (Darquié et al., 2001; Kohno et al., 2009; Le Bihan et al., 2006). Unlike BOLD and IVIM fMRI, which depend primarily on neurovascular coupling, DfMRI was proposed to reflect neuromechanical coupling: rapid changes in water diffusion within neural tissue caused by biophysical events accompanying neuronal activation. Earlier animal studies had already shown diffusion decreases during intense neuronal activation (Zhong et al., 1993, 1997), and these were linked to cell swelling and extracellular-space shrinkage (Dietzel et al., 1980; Latour et al., 1994; Hansen & Olsen, 1980; Hasegawa et al., 1995). Similar activation-related cell-size changes were also observed in brain slices, where vascular confounds are absent (Buckley et al., 1999; Flint et al., 2009).

The neuromechanical-coupling concept rests on a broad body of work showing that physiological activation involves not only electrical and biochemical events but also rapid physical changes in cells and their surroundings. Neuronal activation is accompanied by transient swelling of neurons, dendritic spines, and axons, as well as cytoskeletal rearrangements (Andrew & MacVicar, 1994; Cohen et al., 1968; El Hady & Machta, 2015; Iwasa & Tasaki, 1980; Iwasa et al., 1980; Kim et al., 2007; MacVicar & Hochman, 1991; Tasaki, 1999; Tasaki & Byrne, 1990), together with extracellular-space modulation driven by depolarization, ion fluxes, and osmotic shifts (Dietzel et al., 1980; Holthoff & Witte, 1996; Syková, 2004). Given the high density of synapses (8 10^8^/mm^3^), dendrites (400 m/mm^3^), and cellular processes in cortex (Shapson-Coe et al., 2024), even subtle activity-related dendritic-spine “twitching” (Crick, 1982) may alter the diffusion environment of water molecules (Halpain, 2000; Le Bihan, 2007). Diffusion MRI, therefore, raised the possibility of sensing rapid changes in the intrinsic physical state of neural tissue itself (Fig. 6) (Le Bihan, 2026).

At the same time, DfMRI remained controversial because in vivo diffusion-weighted signals can still contain vascular contributions (Miller et al., 2007). T2-weighted spin-echo diffusion MRI retains residual vascular effects related to BOLD (Aso et al., 2009; de Riedmatten, Spencer, Olszowy, et al., 2025; Kershaw et al., 2009; Miller et al., 2007). There are also IVIM effects (see above) that are mostly visible at low b-values and can be eliminated using higher b-values (Le Bihan et al., 2006). T2-related effects (which are weaker than T2*-weighted BOLD) are largely independent of b-value, so that, when echo time is matched, their contributions can be substantially reduced or eliminated by calculating an ADC or, better, sADC derived from two b-values (Aso et al., 2009; de Riedmatten, Spencer, Olszowy, et al., 2025; Le Bihan et al., 2006; Spencer et al., 2025). Once these confounds are carefully controlled, an intrinsic diffusion response function should be used in place of the canonical hemodynamic response function used in BOLD fMRI (Aso et al., 2009).

Preclinical work has provided strong evidence that DfMRI responses are mechanistically distinct from BOLD responses (Abe, Tsurugizawa, et al., 2017; Tsurugizawa et al., 2013). Diffusion responses are more spatially restricted than gradient-echo BOLD, persist when neurovascular coupling is pharmacologically suppressed while neuronal activity is preserved, and are reduced by interventions that inhibit cell swelling without substantially affecting BOLD (Komaki et al., 2020). Changes in ADC at cellular and tissue level upon pharmacological stimulation have been directly observed using in vivo microscopic diffusion MRI at neuronal level (Abe, Van Nguyen, et al., 2017), Together, these results support the view that DfMRI reflects cellular rather than vascular processes and more directly neuronal functional state. They also motivate a two-stage model of activation in which rapid tissue-microstructural events precede the slower hemodynamic phase measured by conventional fMRI.

DfMRI may, therefore, offer greater spatial and temporal precision than BOLD, including potential sensitivity in white matter, where BOLD fMRI is challenging (de Riedmatten, Spencer, Nguyen-Duc, et al., 2025; Nguyen-Duc et al., 2025; Spencer et al., 2025). Because part of its nonvascular mechanism, it may be less blurred by vascular architecture supplying multiple functional units, as observed with BOLD (Sirotin & Das, 2009). This raises the prospect of resolving finer-scale organization, such as cortical layers or closely interwoven circuits, and of probing faster neural dynamics (Nunes et al., 2021). Beyond these potential practical advantages, DfMRI offers a different view of brain function. Because water homeostasis and intra-/extracellular exchange are central to neuronal physiology, especially in neurons with limited volume regulation (Agre, 2005), activity-related diffusion changes may reflect an active component of signaling rather than merely a by-product. This raises the possibility that rapid mechanical interactions between neighboring cells, such as dendritic-spine mechanics, contribute to information transfer, making diffusion MRI a unique complementary probe of brain activation, offering insights that complement electrophysiology and conventional functional imaging (Le Bihan, 2007).

More broadly, DfMRI is conceptually possible precisely because the tissue of interest is neural tissue and because the field’s prior success with stroke had already shown that diffusion can register rapid pathophysiological changes before gross anatomy shifts. In other words, brain diffusion MRI had already taught investigators that water mobility is sensitive to dynamic cellular states. Functional diffusion MRI asks whether that sensitivity can be extended from pathology into physiology (Le Bihan et al., 2006; Tsurugizawa et al., 2013). The debate around DfMRI also clarifies what counts as evidence in diffusion MRI more generally. To claim direct neural specificity, one must show not only a reproducible signal but also convincingly dissociate it from hemodynamic and other confounds. Animal models, pharmacological perturbations, ultrahigh-field systems, and improved diffusion-encoding schemes have been crucial to this effort.

Whether DfMRI becomes a routine human tool or remains a specialized research method, its importance lies in a broader conceptual insight. DfMRI underscores that diffusion measurements are not limited to static properties of tissue architecture. They can, in principle, reflect dynamic changes in the same architecture, even if those changes are subtle and transient. This reinforces the central thesis that diffusion MRI of the brain occupies a distinctive middle ground between anatomy and physiology (Le Bihan, 2007). It is not merely a structural map, yet it is not a direct electrophysiological readout either. Its future may lie precisely in exploiting that intermediate position intelligently.

### 8.2 A global picture for the brain connectome

Functional MRI, whether BOLD- or diffusion-based, and tractography provide complementary views of the functional and anatomical networks supporting cognition (Gong, He, et al., 2009; Koch et al., 2002). However, tractography primarily depicts large white-matter bundles, poorly resolves intracortical connections, and cannot directly determine the direction, timing, or functional status of information flow. Noninvasively tracking neural activity along specific pathways, therefore, remains challenging. Diffusion MRI can contribute to provide model-dependent estimates of axonal microstructural features. For instance, the g-ratio (inner axonal diameter to outer fiber diameter) is an indirect indicator of relative myelination, which varies widely across axons, and scales with action propagation velocity, from approximately 0.3 m/s in small/short unmyelinated axons to more than 100 m/s in large/long myelinated fibers (Waxman & Bennett, 1972). In combination with other measurements and explicit modeling assumptions, such features may eventually support estimates of conduction velocity (Drakesmith et al., 2019). Such velocity maps could ultimately help estimate propagation delays (Mancini et al., 2021) and cortical synchronization, with potential relevance to psychiatric disorders and consciousness.

**Fig. 7.**
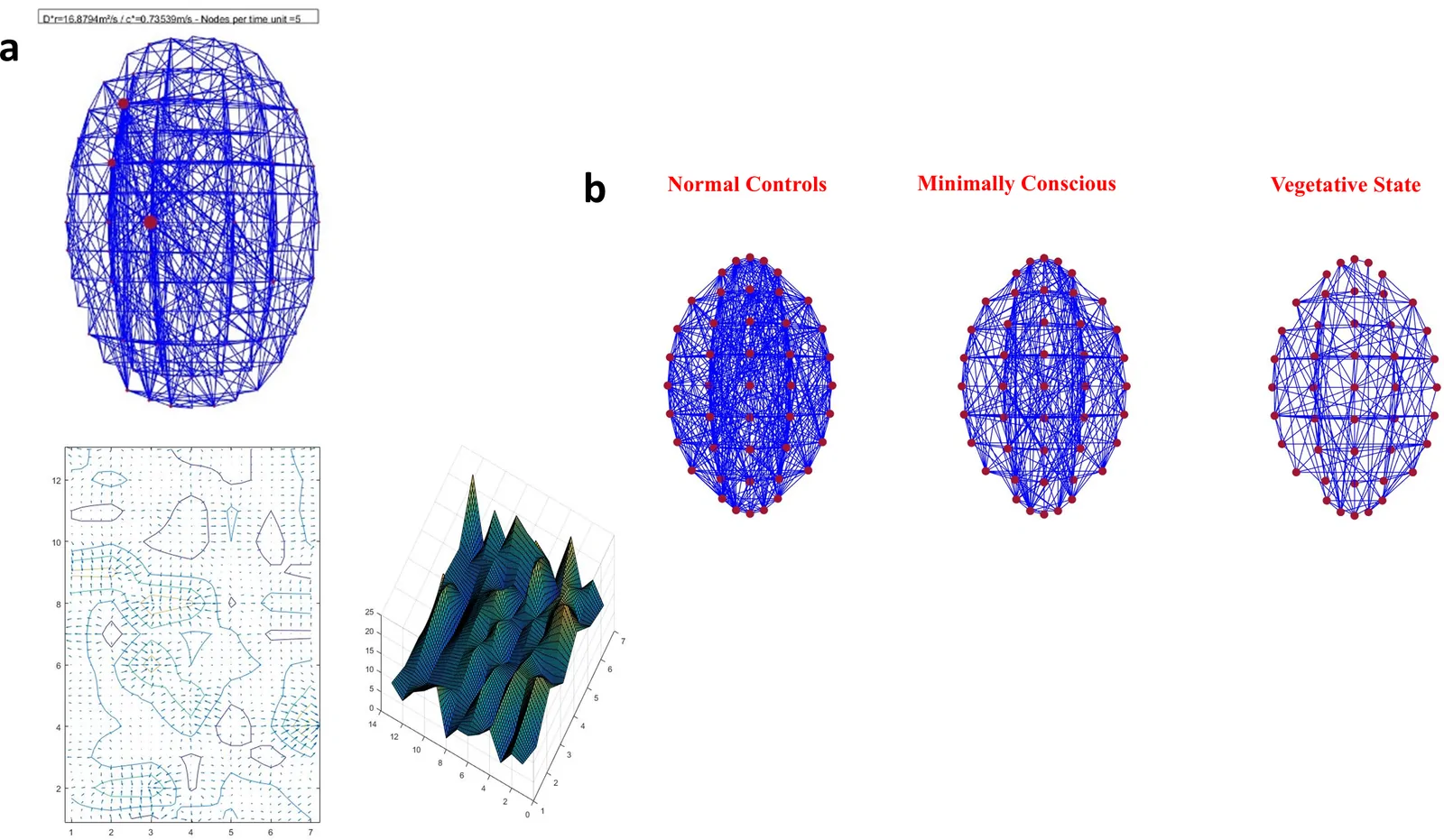
Brain 4D spacetime framework. (a) Propagation of neural activity along fiber tracts (“edges”) between brain nodes can be modeled using an IVIM, relativistic pseudo-diffusion model (pseudo-diffusion coefficient D*) in a 4D brain spacetime with a speed limit c*. Each node is associated with a “mass” depending on its activity that curves the brain connectome 4D spacetime. Activity flow just follows the corresponding geodesics (minimizing distances in this four dimensions spacetime). Reciprocally, activity flow modifies the degree of activation of the nodes and, hence its curvature, changing the spacetime “landscape” dynamically according to mental states. (b) While conscious states are associated with dense, short range, slow speed connections, minimally conscious and vegetative states result from a reduced connectivity based on long range, fast speed connections. Restoring consciousness is tantamount to restoring the curvature of the connectome spacetime, for instance by stimulation of specific key nodes, such as the thalamic nuclei.

The “brain relativistic spacetime” framework is a theoretical proposal that seeks to unify connectome structure and function within a four-dimensional system shaped by finite conduction delays (Le Bihan, 2020, 2023, 2024b). It does not imply that neural signals approach the speed of light, obviously; rather, it adapts elements of Einstein’s relativistic formalism using a brain-specific limiting velocity. The framework first treats action-potential propagation through the connectome as a pseudo-random process, analogous in spirit to the earlier IVIM framework for capillary blood flow. However, because neural propagation has a finite maximum speed within the connectome, a relativistic formalism using a brain-specific limiting velocity is required, which is not trivial matter (Le Bihan, 2020). As a consequence, space and time become interdependent, making the functional connectome analogous to a four-dimensional spacetime. This spacetime is naturally flat, but becomes functionally curved by distributed neuronal activity (nodes) that are modulating short- and long-range connections (edges), just as mass–energy curves the physical spacetime in General Relativity. Hence, this framework further suggests a gravity-like role for neural activity and consciousness, which, obviously, requires empirical testing. Still, it has been used to interpret faulty connections revealed by DTI in relation to psychiatric phenotypes, including hallucinations in schizophrenia (Le Bihan, 2020), delayed conscious access in schizophrenia and bipolar disorder (Berkovitch et al., 2021), and depressive symptoms associated with default-mode and salience network hyperactivity involving poorly myelinated-slow conducting fibers (Mulders et al., 2015; Wang et al., 2024). During anesthesia or minimally conscious states, reduced thalamic activity could be interpreted within this framework as a “flattening” of functional geometry and a weakening of effective connectivity, despite residual local activity, as revealed through DTI and resting-state fMRI (Fig. 7) (Demertzi et al., 2019). Such a situation may characterize vegetative states, in which patients appear awake without signs of awareness (Owen et al., 2006). In anesthetized rats, central–medial thalamic stimulation induced awakening and was associated with local and distributed ADC reductions that, within this framework, could be interpreted as a restoration of connectivity with cortical areas and, hence, of the connectome spacetime “curvature” (Abe, Tsurugizawa, et al., 2017). Related findings have been reported in nonhuman primates (Tasserie et al., 2022) and in patients with disorders of consciousness treated with deep brain stimulation (Warren et al., 2025), although substantial technical and ethical challenges remain, emphasizing the potential interest of this theoretical framework.

### 8.3 Diffusion-based virtual MR elastography

Magnetic resonance elastography (MRE) (Muthupillai et al., 1995) has established that the brain is a viscoelastic organ whose stiffness varies across regions, disease states, and age. Brain elasticity can, therefore, be viewed as a mechanobiological marker of tissue microstructure, reflecting the integrated contribution of dendrites, axons, astrocytic processes, extracellular matrix, and interstitial-water balance. White-matter disorganization, myelin loss, neuritic fragmentation, and extracellular remodeling revealed by diffusion MRI may all contribute to a softer and more dissipative tissue response. Regional stiffness maps may thus provide a mesoscale index of cumulative microstructural failure and of how strongly pathology has destabilized the local tissue environment (Coelho & Sousa, 2022; Y. Feng et al., 2024; Le Bihan, 2026). Conventional MRE is noninvasive, but it generally relies on externally induced mechanical waves to estimate shear stiffness (Muthupillai et al., 1995). Such vibrations may be undesirable in some vulnerable populations. Weaver and colleagues showed that intrinsic brain motion generated by cardiac pulsatility can also be exploited to estimate mechanical properties (Weaver et al., 2012; Zorgani et al., 2015). Still, whether externally or intrinsically driven, MRE remains constrained by skull attenuation, reflection, diffraction, limited spatial coverage, and wavelength-dependent spatial resolution (Low et al., 2016).

**Fig. 8.**
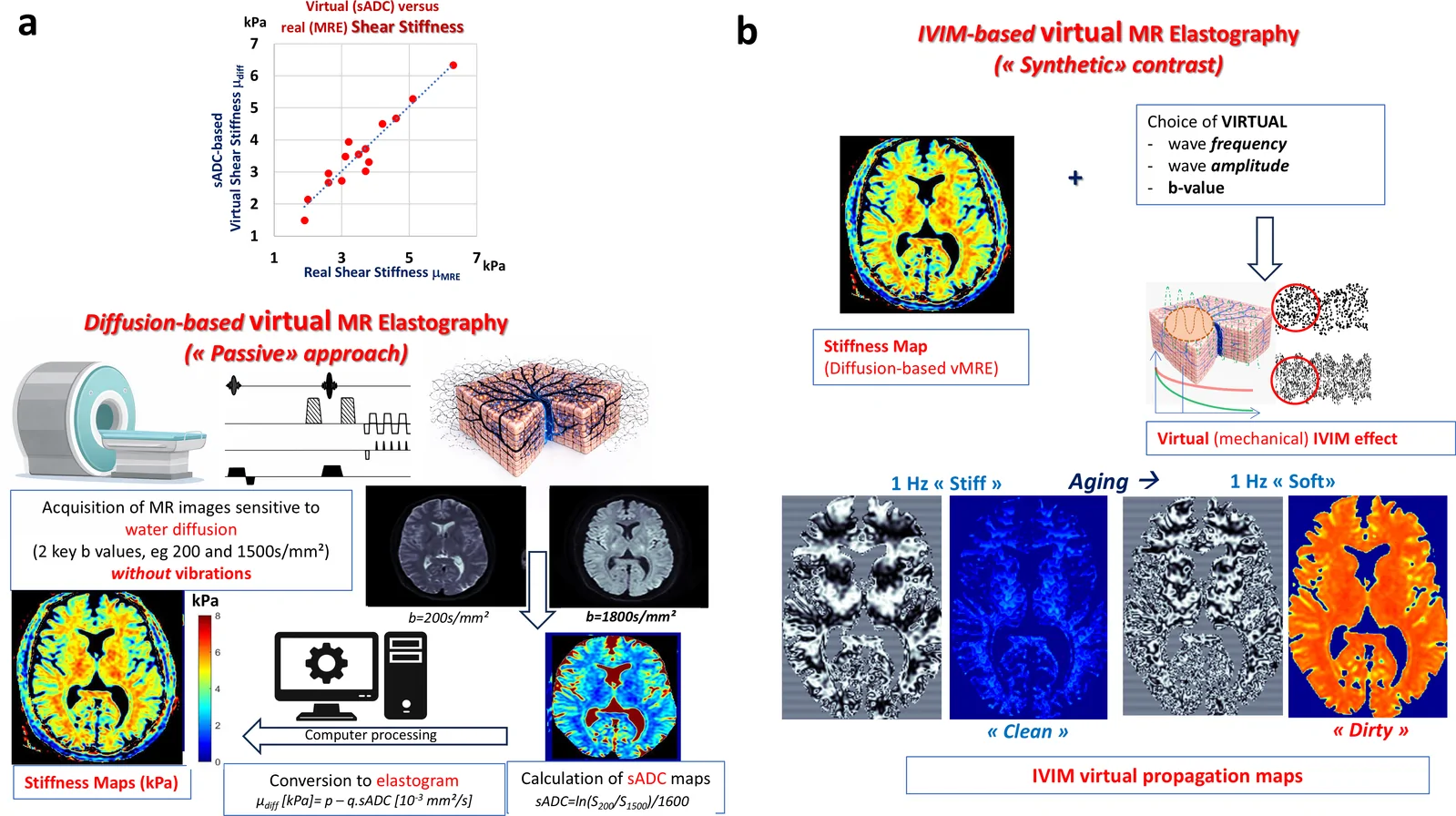
Diffusion-based virtual MR elastography. (a, top) There is a strong linear relationship between sADC-based and MRE-based shear stiffness, as shown here in the liver as both markers are linked to tissue microstructure. Using this linear relationship, the sADC can be converted to shear stiffness. (a, bottom) Diffusion-based virtual MRE: Diffusion-weighted images are acquired at two key b-values (e.g., 200 and 1,800 s/mm²) to calculate sADC maps. The sADC maps are then converted to elastograms (quantitative shear-stiffness maps) using a linear relationship between sADC and shear stiffness. (b) IVIM-based virtual MRE: The process is reversed, using the known stiffness maps (either from standard MRE or from diffusion-based MRE) the propagation of virtual waves is simulated. Mechanical (virtual) waves propagating in a tissue induce a magnetization phase shift along the direction of a motion-probing Gradient pulse. This phase shift presents a spatial distribution that results in a IVIM effect within each voxel, with a signal amplitude attenuation that depends on the voxel stiffness. Softer tissues exhibit shorter wavelengths and thus higher intravoxel motion dispersion, leading to high signal attenuation. Stiffer tissues exhibit less attenuation. IVIM-based vMRE allows to generate a new type of image contrast based on elasticity properties for any choice of (virtual) wave frequency or amplitude. In the normal brain, IVIM-vMRE suggests that, due to its high stiffness, propagating wavelength induced by cardiac pulsations (about 1 Hz) is about the size of the brain, hence potentially optimizing clearance of proteins by the glymphatic system. With aging brain, stiffness decreases and the brain parenchyma becomes “softer”, resulting in lower propagation speed and shorter wavelength, potentially making the glymphatic system less efficient to “clean” the brain.

Diffusion-based virtual MRE (vMRE) offers a potential alternative by inferring tissue stiffness from diffusion MRI without requiring physical shear-wave propagation (Le Bihan et al., 2017). Its rationale is that microstructural features that hinder water diffusion, such as cellularity, axonal packing, myelination, extracellular-matrix organization, and tissue geometry, also contribute to mechanical stiffness. Using optimized diffusion metrics such as shifted apparent diffusion coefficient (sADC), which is more sensitive to tissue microstructure than conventional ADC (see above) and that has been shown to be linearly and negatively correlated with shear stiffness, diffusion-based vMRE can generate quantitative stiffness estimates in kPa (Fig. 8) (Le Bihan et al., 2017). This approach was first evaluated and validated for liver-fibrosis staging (Kromrey et al., 2020; Le Bihan et al., 2017).

IVIM-based vMRE further expands this concept by generating virtually synthesized rather than physically induced shear-wave phenomena within tissues using the principles of IntraVoxel Incoherent Motion (IVIM) established earlier for blood microcirculation (Le Bihan, 2019; Le Bihan et al., 2017) to model the intravoxel phase-shift distribution that would be induced by virtual tissue motion. This development enables an entirely new class of elastographic contrast, one not constrained by mechanical systems, vibration frequencies, driver placement, or tissue-wave attenuation physics. In conventional MRE, amplitude and frequency of real shear waves are dictated by hardware and human tolerances. Try to push too high a frequency, and waves fail to propagate; too low, and measurement precision drops. In IVIM-based virtual elastography, wave propagation is no longer a physical event—it becomes a *simulated* behavior, inferred from the relationship between tissue stiffness, as obtained through diffusion-vMRE, and the attenuation of the amplitude of motion-encoded MR signals induced by a virtual traveling wave (Fig. 8). This conceptual evolution redefines elastography as a computational transformation of diffusion data, extending elasticity imaging into a domain of virtual mechanics set by user-defined parameters that could reveal pathological heterogeneity not visible with conventional imaging (Le Bihan et al., 2017).

For brain clinical applications, diffusion-vMRE may be technically simpler and more deployable than conventional MRE. Unlike MRE, vMRE does not require external mechanical vibrations, which may be difficult to use in selected settings, particularly patients with traumatic brain injury, elderly population or children (vMRE is even used in obstetrics (Deng et al., 2023)). Mechanical stiffness is recognized by neurosurgeons as relevant to approach selection, resection strategy, and complication risk (Lagerstrand et al., 2021). An early neurosurgical application is the preoperative assessment of pituitary–adenoma consistency. In patients undergoing transsphenoidal surgery, preoperative vMRE stiffness and elasticity-histogram metrics correlated with surgeons’ intraoperative impressions of tumor softness or firmness, with good feasibility and reproducibility (Lagerstrand et al., 2021). Because consistency directly affects whether a tumor is suctionable or fibrous, this information may improve surgical planning. Meningioma surgery is another potential indication because tumor firmness is difficult to predict on conventional MRI. vMRE-derived stiffness was associated with intraoperative consistency and heterogeneity, with higher stiffness associated with harder tumors and greater stiffness variation linked to more heterogeneous lesions (Aunan-Diop et al., 2023). More recently, vMRE showed strong agreement with conventional MRE across gliomas, meningiomas, and schwannomas while also correlating with intraoperative grading and clinicopathological features (He et al., 2025). Further advances are expected with the assessment of anisotropic elasticity, separating radial and axial stiffness, which would require using the DTI framework with two key b values, to obtain “shifted” radial and axial diffusivities.

Diffusion-based virtual MRE also appears as an attractive, scalable noninvasive biomarker to investigate brain stiffness in the aging brain and for longitudinal or interventional studies of neurodegenerative diseases, such as Alzheimer’s disease including intervention stratification (Le Bihan, 2026). In healthy aging, most studies report progressive brain softening, which is associated with lower cognitive performance (Coelho & Sousa, 2022; Y. Feng et al., 2024; Hiscox et al., 2018). Altered mechanical properties have also been associated with cognitive decline and occurrence of neurodegenerative diseases (Pavuluri et al., 2025; Schwarb et al., 2016). In the normal brain, the cardiac frequency (about 1 Hz) is the nominal frequency that results in heart-induced mechanical waves to propagate to about 20 cm/s, so that each heart beat propagates to the whole brain. When elasticity decreases with age, the propagation speed goes down, so that cardiac pulsations may become less efficient to “clean” the brain (Fig. 8) through the glymphatic system, a CSF–interstitial fluid transport pathway, supported by perivascular spaces, astrocytic water channels, vascular pulsatility, and meningeal lymphatic drainage, that contributes to waste clearance and fluid homeostasis in the brain (Iliff et al., 2012). Standard diffusion MRI and DTI do not directly image glymphatic transport in the same way that tracer studies do, but can probe free-water, CSF flow (Le Bihan et al., 1987), and perivascular fluid movement (Harrison et al., 2018), perivascular spaces and adjacent tissue anisotropy, as illustrated by DTI-ALPS (Naganawa & Taoka, 2022), although this method has been found nonspecific with results to be interpreted cautiously (Taoka et al., 2026). Diffusion-vMRE could provide a surrogate marker of parenchymal mechanics relevant to glymphatic function, allowing from individual brain elasticity values to assess vulnerability to Alzheimer’s disease and potentially identify patients who might benefit from early interventions (Le Bihan, 2026).

Interestingly, cortical stiffness estimated through conventional MRE (functional MRE) has been reported to transiently increase during neuronal activation induced by sensory or motor stimulation, suggesting that viscoelastic neural properties are modulated by physiological activation. In humans, visual stimulation increased stiffness in visual cortex by about 6–11% (Lan et al., 2020), mirroring activation-related ADC and sADC decreases observed with DfMRI (Le Bihan et al., 2006). Preclinical work suggests that some biomechanical changes occur faster than BOLD responses, in a similar way to DfMRI (Nunes et al., 2021; Patz et al., 2019; Tsurugizawa et al., 2013). These observations are consistent with the known negative relationship between shear stiffness and sADC, confirming that shear stiffness and sADC are, in some way, interchangeable or dual markers of dynamic microstructure.

## 9 Limits, Pitfalls, and Future Directions

The maturity of brain diffusion MRI brings with it a clear view of its limits. The first is biological nonspecificity. Metrics such as ADC, FA, mean diffusivity, kurtosis, or model-derived intracellular fractions are not self-interpreting. They summarize the behavior of water under a particular encoding scheme and must be linked to anatomy and pathology through models that are always incomplete. The same change in a scalar metric can arise from multiple microstructural causes. Brain diffusion MRI is, therefore, strongest when used comparatively and contextually rather than as a stand-alone tissue assay (Beaulieu, 2002; Novikov et al., 2019).

The second limit is geometric complexity. Crossing fibers, cortical folding, partial volume with CSF, and lesions that distort anatomy all challenge inference. White-matter tractography is powerful precisely because the brain has coherent bundles, but those same bundles intersect, diverge, and curve in ways that frustrate simplistic reconstruction. Higher angular-resolution methods, better distortion correction, and anatomically informed tractography improve matters, yet they do not eliminate the inverse problem (Daducci et al., 2016; Tournier et al., 2011).

The third limit is validation. Histological comparison is difficult because diffusion measurements average over living-tissue properties, fixation changes microstructure, and one-to-one registration across scales is imperfect. Model validation, therefore, requires triangulation among ex vivo microscopy, animal studies, simulations, test–retest analysis, pathology-specific hypotheses, and increasingly multimodal integration. The brain field has made major progress here, but the lesson is sobering: the more ambitious the biological claim, the stronger the need for external validation (Jelescu & Budde, 2017; Novikov et al., 2019).

On the technical side, several directions are especially promising. Stronger gradients and more flexible diffusion encoding allow sampling of previously inaccessible regimes, improving sensitivity to small compartments and time dependence. Better motion and distortion correction are crucial for brain applications, especially near the skull base and in pediatric or patient populations. Higher spatial resolution will reduce partial-volume confounds in cortex and small tracts. Ultrafast acquisitions may stabilize diffusion fMRI and other dynamic paradigms. Free-water correction, multi-shell acquisition, and joint structural–functional modeling are already making diffusion MRI less reliant on a few oversimplified scalar summaries (Assaf et al., 2019; Fieremans et al., 2016).

Conceptually, the future of brain diffusion MRI likely lies in integration rather than isolation. Diffusion data can provide the structural and microdynamic substrate, while relaxometry, susceptibility imaging, myelin-sensitive methods, spectroscopy, perfusion imaging, electrophysiology, and functional MRI supply complementary information. For connectomics, structural pathways inferred from diffusion should be considered together with conduction delays, synaptic physiology, and dynamic network behavior. For disease, diffusion abnormalities become most interpretable when read alongside clinical phenotype, longitudinal evolution, and other imaging contrasts. This pluralistic approach is not a retreat. It is the mature recognition that water diffusion is profoundly informative but never exhaustive.

The brain will likely remain diffusion MRI’s most demanding and rewarding arena. It contains the clearest examples of early clinical utility, the most intricate organized microstructure, and the most ambitious interpretive goals. The field began by asking whether MRI could image diffusion at all; it now asks whether diffusion MRI can help estimate axon caliber and conduction velocity, cortical microarchitecture, fluid transport, exchange, and even neural activation. That trajectory is the route by which a specialized pulse-sequence concept became one of the major quantitative languages of modern neuroscience and neurological imaging.

## 10 Conclusion

Diffusion MRI is powerful for investigating normal and diseased brain tissue because water motion is strongly constrained by cellular and mesoscale architecture. Its success in stroke, white-matter mapping, tractography, and microstructure imaging nonetheless imposes discipline: diffusion is a rigorously encoded measurement of displacement statistics, not a pictorial surrogate for histology. Biological interpretation must pass through pulse-sequence physics, model assumptions, and the finite identifiability of inverse problems.

Thus, the mature view of the field is both ambitious and cautious. Diffusion MRI can reveal abnormalities before gross anatomy changes, map anisotropy and pathway organization noninvasively, and provide access to non-Gaussian, time-dependent, and multi-compartment behavior. Yet no scalar map or tractogram is self-interpreting. Stronger encoding, better models, careful validation, and multimodal integration are needed to turn sensitive phenomena into reliable microstructural and functional inference.

## Data Availability

There are no data or code associated with this article.
